# A novel approach for large-scale manufacturing of small extracellular vesicles from bone marrow-derived mesenchymal stromal cells using a hollow fiber bioreactor

**DOI:** 10.3389/fbioe.2023.1107055

**Published:** 2023-01-24

**Authors:** Viktoria Jakl, Melanie Ehmele, Martina Winkelmann, Simon Ehrenberg, Tim Eiseler, Benedikt Friemert, Markus Thomas Rojewski, Hubert Schrezenmeier

**Affiliations:** ^1^ Institute for Transfusion Medicine, University Hospital Ulm, Ulm, Germany; ^2^ Institute for Clinical Transfusion Medicine and Immunogenetics Ulm, German Red Cross Blood Donation Service Baden-Württemberg—Hessia and University Hospital Ulm, Ulm, Germany; ^3^ Clinic of Internal Medicine I, University Hospital Ulm, Ulm, Germany; ^4^ Clinic for Trauma Surgery and Orthopedics, Army Hospital Ulm, Ulm, Germany

**Keywords:** mesenchymal stromal cells, mesenchymal stem cells, platelet lysate, hollow fiber bioreactor, exosomes, small extracellular vesicles, isolation

## Abstract

Mesenchymal stromal cells (MSCs) are promising therapeutic candidates in a variety of diseases due to having immunomodulatory and pro-regenerative properties. In recent years, MSC-derived small extracellular vesicles (sEVs) have attracted increasing interest as a possible alternative to conventional cell therapy. However, translational processes of sEVs for clinical applications are still impeded by inconsistencies regarding isolation procedures and culture conditions. We systematically compared different methods for sEV isolation from conditioned media of *ex vivo* expanded bone marrow-derived MSCs and demonstrated considerable variability of quantity, purity, and characteristics of sEV preparations obtained by these methods. The combination of cross flow filtration with ultracentrifugation for sEV isolation resulted in sEVs with similar properties as compared to isolation by differential centrifugation combined with ultracentrifugation, the latter is still considered as gold standard for sEV isolation. In contrast, sEV isolation by a combination of precipitation with polyethylene glycol and ultracentrifugation as well as cross flow filtration and size exclusion chromatography resulted in sEVs with different characteristics, as shown by surface antigen expression patterns. The MSC culture requires a growth-promoting supplement, such as platelet lysate, which contains sEVs itself. We demonstrated that MSC culture with EV-depleted platelet lysate does not alter MSC characteristics, and conditioned media of such MSC cultures provide sEV preparations enriched for MSC-derived sEVs. The results from the systematic stepwise evaluation of various aspects were combined with culture of MSCs in a hollow fiber bioreactor. This resulted in a strategy using cross flow filtration with subsequent ultracentrifugation for sEV isolation. In conclusion, this workflow provides a semi-automated, efficient, large-scale-applicable, and good manufacturing practice (GMP)-grade approach for the generation of sEVs for clinical use. The use of EV-depleted platelet lysate is an option to further increase the purity of MSC-derived sEVs.

## Introduction

Within the last decades, the interest in mesenchymal stromal/stem cells (MSCs) increased continuously due to their regenerative and immunomodulatory potential. MSCs were first identified by Friedenstein et al. in 1976 as fibroblast precursors (Friedenstein et al., 1976). Since then, a lot of research was performed, and in 2006, the International Society for Cellular Therapy (ISCT) proposed minimal criteria for the definition of MSCs (Dominici et al., 2006). These included plastic adherence of MSCs when being cultured under standard culture conditions, expression of surface antigens cluster of differentiation (CD) 73, CD90, and CD105, and lack of expression of common leukocyte and hematopoietic cell markers (e.g., CD45, CD34, CD14, CD11b, CD79α, or CD19 and histocompatibility leukocyte antigen (HLA) DR) and differentiation capacity into cells of the three mesenchymal lineages (adipocytes, chondrocytes, and osteoblasts) (Dominici et al., 2006). MSCs can be found in numerous tissues of the human body such as bone marrow (BM), adipose tissue, umbilical cord, or dental pulp.

The therapeutic potential of BM-derived MSCs has been shown in a variety of clinical applications like in bone regeneration (Soler et al., 2016; Gjerde et al., 2018; Gomez-Barrena et al., 2019; Gomez-Barrena et al., 2020; Gomez-Barrena et al., 2021) or wound healing (Falanga et al., 2007; Lataillade et al., 2007; Yoshikawa et al., 2008; Dash et al., 2009; Lu et al., 2011), and today, more than 1,200 clinical trials investigating MSC therapy are listed at clinicaltrials.gov (for search term “mesenchymal stromal cells” or “mesenchymal stem cells,” retrieved 11/22/2022). The application of MSCs to humans as an advanced therapy medicinal product (ATMP) has been proven to be safe. However, there are concerns regarding genetic stability (Pan et al., 2014; Stultz et al., 2016), replicative senescence (Wagner et al., 2008), and promotion of tumor proliferation (Pavon et al., 2018) when using *ex vivo* expanded MSCs. Therefore, in recent years, MSC-derived factors such as extracellular vesicles (EVs) became increasingly popular as therapeutic effectors. EVs are membrane-surrounded particles that are secreted by various cell types and are important drivers of intercellular communication by exchanging their cargo (e.g., nucleic acids, lipids, and proteins), thereby modulating different molecular events in the recipient cells. EVs can be subdivided into three main groups, apoptotic bodies (which arise from dying cells during apoptosis), microvesicles, and exosomes. While microvesicles directly bud from the plasma membrane and can be up to 1,000 nm in size, exosomes are released into the extracellular space from intracellular multivesicular bodies and range from 40 to 100 nm in diameter (Raposo and Stoorvogel, 2013). As there are no unique markers for discrimination between different subsets of EVs, Théry et al. proposed the term of small extracellular vesicles (sEVs) for EVs with sizes smaller than 200 nm instead of referring them after their origin (e.g., exosomes) (Thery et al., 2018). As compared to classical cell therapy, sEVs show several advantages including their potential to cross biological barriers (e.g., blood–brain barrier) (Banks et al., 2020), ease of sterilization (e.g., by filtration) (Elsharkasy et al., 2020), and their non-viable nature (due to lack of a functional nucleus) (Thery et al., 2018). Studies directly comparing MSCs and MSC-derived mediators showed similar or even improved therapeutic effectiveness for the latter (Bruno et al., 2009; Shao et al., 2017).

Differential centrifugation (DC) with final enrichment of sEVs by ultracentrifugation (UC) still represents the most commonly used procedure for isolation of sEVs (Gardiner et al., 2016). However, being labor-intensive and time-consuming, centrifugation-based strategies alone were assumed not to be suitable for large-scale purification of sEVs (Lener et al., 2015; Zeringer et al., 2015; Gurunathan et al., 2019; Witwer et al., 2019). Hence, multi-step approaches combining several methods for initial volume reduction and concentration followed by final sEV enrichment became increasingly popular (Gardiner et al., 2016). Ultrafiltration such as cross flow filtration (CFF) through membranes with different pore sizes or polymer-based methods such as precipitation with polyethylene glycol (PEG) can be applied for concentrating the volume of the starting material (Coumans et al., 2017), although precipitation has been shown to result in sEV preparations with reduced purity (Van Deun et al., 2014; Lobb et al., 2015). For final purification of sEVs, size exclusion chromatography (SEC) could be used besides UC. However, small input volumes for SEC columns limit their use for large-scale purification required for clinical applications (Busatto et al., 2018; Paganini et al., 2019).

Contamination with sEVs from sources other than MSCs can occur due to serum-containing cell culture supplements such as platelet lysate (PL) (Witwer et al., 2019; Almeria et al., 2022). Although sEVs from different sources can be discriminated by surface antigen expression such as lacking expression of CD81 on PL-derived sEVs (Koliha et al., 2016; Wiklander et al., 2018), downstream separation of contaminating sEVs would be difficult. Therefore, collecting sEVs during a starvation period with serum-free or EV-depleted cell culture supplements is commonly applied. Since changed sEV profiles appeared as a consequence of switching to serum-free culture media (Li et al., 2015; Haraszti et al., 2019), and PL-derived sEVs were found to be taken up by MSCs (Torreggiani et al., 2014), absence of PL-derived sEVs could impact characteristics of MSC sEVs due to suboptimal cell expansion conditions (Borger et al., 2020), and cellular stress (Wiest and Zubair, 2020; Almeria et al., 2022).

Adlerz et al. assumed conditioned media (CM) of about 500 million cells to be a requisite for sEV numbers necessary for clinical applications (Adlerz et al., 2020). Large amounts of starting material are hard to generate in conventional cell culture, hence, large-scale expansion methods such as hollow fiber bioreactors could help in resolving this problem by allowing large-scale expansion of MSCs (Rojewski et al., 2013) and the production of several liters of CM (Watson et al., 2016). The Quantum® Cell Expansion System from Terumo BCT (Quantum system) comprises a hollow fiber bioreactor attached to several tubings connected with bags for fluidics in- and output. Being a single use unit, the expansion set is loaded into an incubator with pumps, valves, gas inlet, and user interface allowing for semi-automated expansion of cells. The bioreactor itself is composed of approximately 11,000 hollow fibers providing a growth surface of up to 21,000 cm^2^ after coating with proteins such as cryoprecipitate (CP) that enable attachment of cells.

The number of pre-clinical studies investigating sEVs as an MSC substitute increases continuously; however, only few of them have gone across experimental animal models toward a clinical application (36 studies listed at clinicaltrials.gov for search term “mesenchymal stromal cells AND exosomes” or “mesenchymal stem cells AND exosomes” or “mesenchymal stromal cells AND extracellular vesicles” or “mesenchymal stem cells AND extracellular vesicles,” retrieved: 11/22/2022). This could be in part explained by a high burden in the translational process from laboratory-scale protocols toward large-scale manufacturing of clinical doses as standardized and universal procedures have not been established yet. In addition, sEV characteristics change upon alteration of purification (e.g., isolation method) and culture strategies (e.g., growth media and expansion system), respectively (Lener et al., 2015; Doyle and Wang, 2019; Adlerz et al., 2020; Gowen et al., 2020; Wiest and Zubair, 2020). During this study, suitability of several sEV isolation methods were evaluated prior to an implementation of a hollow fiber bioreactor-based expansion process enabling the large-scale manufacturing of sEVs.

Here, we use this established tool box of various sEV isolation methods and perform a head-to-head comparison to gold standard method DC combined with UC using the starting material of same donors in order to exclude variability from MSC lines or their donors as a confounding factor. Furthermore, we combine the MSC expansion and generation of CM in a hollow fiber bioreactor with subsequent steps of sEV isolation in a novel workflow.

## Materials and methods

### Cell culture and collection of CM

#### Harvesting of primary material

Primary MSCs derived from BM aspirates (iliac crest) of healthy volunteer donors were used for the following experiments. Collection of the material has been approved by the Ethical Committee of the University of Ulm (Ulm, Germany) and informed consent was obtained from all donors. Aspiration was performed by following standard operating procedures to obtain a small-volume BM aspirate of approximately 25–35 mL. MSCs of passage 0 (P0) were obtained as previously described in detail (Rojewski et al., 2019). We used MSCs from up to 10 different donors. MSCs from different donors might also differ in the release of sEVs and their properties. Therefore, CM from the same MSC lines have been used for the experiments comparing different sEV isolation methods against gold standard purification by DC combined with UC (except for one of isolation method II) and different expansion systems for sEV generation. Thus, the comparisons reflect the impact of the different isolation methods and are not influenced by the potential variability among MSC donors.

#### Cell expansion for optimization of sEV isolation methods and collection strategies

MSCs were seeded at 2,000–4,000 cells/cm^2^ in αMEM supplemented with 8% PL (IKT Ulm, Ulm, Germany) and 1 i.U. per mL heparin (Ratiopharm GmbH, Ulm, Germany) (αMEM+8% PL) and expanded for passage 1 (P1) or passage 2 (P2). After 24–96 h, the media was exchanged completely for subsequent collection of sEVs in CM. Collection was performed for 24–48 h using either αMEM+8% PL or αMEM+8% EV-depleted PL (EV depl. PL). The cells were harvested using TrypZean™ (Lonza Group Ltd., Basel, Switzerland), and the cell count was determined with a Neubauer chamber (Glaswarenfabrik Karl Hecht GmbH & Co., KG, Sondheim vor der Rhön, Germany). Dead cells were identified by trypan blue staining (Sigma-Aldrich Chemie GmbH, Taufkirchen, Germany), and viability of cells was given by the ratio of living cell count and total cell count. CM was harvested and stored at −80°C until isolation of sEVs.

PL was manufactured as previously described (Fekete et al., 2012a). For the generation of EV depl. PL, PL was ultracentrifuged for 3 h at 120,000 × g at 4°C (Optima™ LE-80K with SW 28 Ti Swinging-Bucket Aluminum Rotor; Beckman Coulter GmbH, Krefeld, Germany), and the supernatant was subsequently sterile filtered through Sartolab® RF vacuum filtration units (Sartorius Lab Instruments GmbH & Co., KG, Göttingen, Germany).

#### Cell expansion for the implementation of a hollow fiber bioreactor

The Quantum® Cell Expansion System (Terumo BCT, Inc., Lakewood, United States) was used for large-scale expansion of MSCs. A two-step expansion process was performed, where MSCs were isolated from BM in a first run (resulting in P0 MSCs; data not shown) followed by a second run, where MSCs were expanded for P1 and sEVs were collected at the end of the run. Briefly, harvested P0 MSCs were stored at room temperature (RT) for approximately 6 h until the preparation of the Quantum system for the next run was completed. This included loading of the single use cell expansion set, priming with phosphate-buffered saline (PBS; Lonza Group Ltd.), coating with CP for 4 h and conditioning of media (αMEM+8% PL) for 1 h. CP was manufactured from fresh frozen plasma (FFP; IKT Ulm) as follows. FFP of 16 different donors (about 300 mL per donor) was thawed at 4°C overnight, pooled, and centrifuged at 4,777 × g for 10 min at RT. Supernatant was discarded, 0.1 mL PBS was added per mL FFP and carefully mixed. After incubation for 1 h at RT, resulting CP stock solution was divided into 7.5-mL aliquots and stored at −20°C. For preparation of CP as coating solution, one aliquot of CP stock solution was thawed at RT and filled up with PBS to a total volume of 100 mL. MSCs were seeded into the bioreactor at 1,000 cells/cm^2^ and were allowed to attach for 24 h. The media was fed continuously, and the flow rate was adapted according to daily measured lactate concentrations in CM ranging from 0.1 mL/min at the beginning of the run to a maximum of 1.6 mL/min. A new waste bag was connected to the Quantum system 16–19 h prior to the end of the run. CM was collected during this time in addition to a complete system flush directly before cell harvest, resulting in total CM volumes of 700–2,000 mL. Duration of Quantum-based cell expansion varied from 6–9 days.

MSCs obtained from the same donor as for the Quantum system were in parallel isolated (P0; data not shown) and expanded for P1 in a conventional cell culture process in CellSTACK® Culture Chambers (CellSTACK; Corning Incorporated, New York, United States) with a surface area of 1,272 cm^2^ as previously described (Fekete et al., 2012b; Rojewski et al., 2019). Briefly, the cells were seeded at 4,000 cells/cm^2^ in αMEM+8% PL and grown for 4–6 days. The media was exchanged completely 24–48 h prior to harvesting of cells, and sEVs were collected in CM.

CM was stored at −80°C until isolation of sEVs for both expansion systems, and the cells were harvested using TrypZean™ (Lonza Group Ltd.). Cell count and viability were determined by trypan blue staining using a Neubauer chamber as described previously.

### Characterization of MSCs

#### Flow cytometric characterization of MSCs

The surface antigen expression of MSCs was identified by flow cytometry using the following antibodies: CD14 (clone HCD14; BioLegend, San Diego, United States or clone MφP9; BD Biosciences, New Jersey, United States), CD34 (clone 8G12 also known as HPCA2), CD45 (clone HI30), CD73 (clone AD2), CD90 (clone 5E10), CD105 (clone 266), and HLA DRDPDQ (clone Tu39 also known as TÜ39) (all from BD Biosciences). The cells were stained as per manufacturer’s instructions (for staining details see Supplementary Table S1) and fluorescence intensities were measured using the FACSCelesta™ Cell Analyzer with BD FACSDiva™ software (BD Biosciences). Surface antigens were subdivided into identity markers (CD73, CD90, and CD105) and purity markers (CD14, CD34, CD45, and HLA DRDPDQ).

#### Differentiation assays

MSCs were differentiated into cells of adipogenic, chondrogenic, and osteogenic lineages by using differentiation assay kits (human mesenchymal stem cell (hMSC), Adipogenic Differentiation Medium BulletKitTM (Lonza Group Ltd.), and StemMACS™ ChondroDiff Media, human and StemMACS™ OsteoDiff Media, human (both from Miltenyi Biotec B.V. & Co., KG, Bergisch Gladbach, Germany)), as per the manufacturer’s instructions. Briefly, the cells were seeded at 4.5–20 × 10^6^ cells/cm^2^, and the media were exchanged every 2—3 days until differentiation of cells was completed. For chondrogenic differentiation, three-dimensional (3D) pellet culture was replaced by a two-dimensional (2D) expansion of cells, as previously performed (Rojewski et al., 2019). The cells grown in αMEM+20% fetal bovine serum (FBS; Biological Industries, Kibbutz Beit Haemek, Israeal) were used as controls. The cells were stained by Oil Red O and hematoxylin (adipogenic differentiation; Sigma-Aldrich Chemie GmbH) and methylene blue (chondrogenic differentiation; Sigma-Aldrich Chemie GmbH), and alkaline phosphatase activity was visualized by the 5-bromo-4-chloro-3-indolylphosphate (BCIP)/nitroblue tetrazolium (NBT) substrate (osteogenic differentiation; Sigma-Aldrich Chemie GmbH), respectively. Microscopic pictures were taken using an inverted phase contrast microscope (BZ-X710; KEYENCE DEUTSCHLAND GmbH, Neu-Isenburg, Germany) with BZ-X Viewer software.

#### Proliferation assay

Proliferation of cells was analyzed by using the CyQUANT™ Cell Proliferation Assay Kit (Thermo Fisher Scientific Inc., Waltham, United States) as per manufacturer’s instructions. Briefly, 200 cells per well were seeded into a 96-well plate as triplicates (*n* = 3) in αMEM+8% PL. After 4 days, the media was exchanged to αMEM without (w/o) additional supplement, αMEM+8% PL and αMEM+8% EV depl. PL, respectively, and the cells were grown for 24 h and 48 h. The cells were washed with PBS, and cell pellets were frozen at −80°C. DNA of lysed cells was stained by CyQUANT™ GR dye, and fluorescence intensities were measured by a microplate reader POLARstar Omega (BMG LABTECH GmbH, Ortenberg, Germany), with Reader Control and MARS Data Analysis software.

### Isolation, quantification, and characterization of sEVs

#### Isolation of sEVs

**FIGURE 1:**
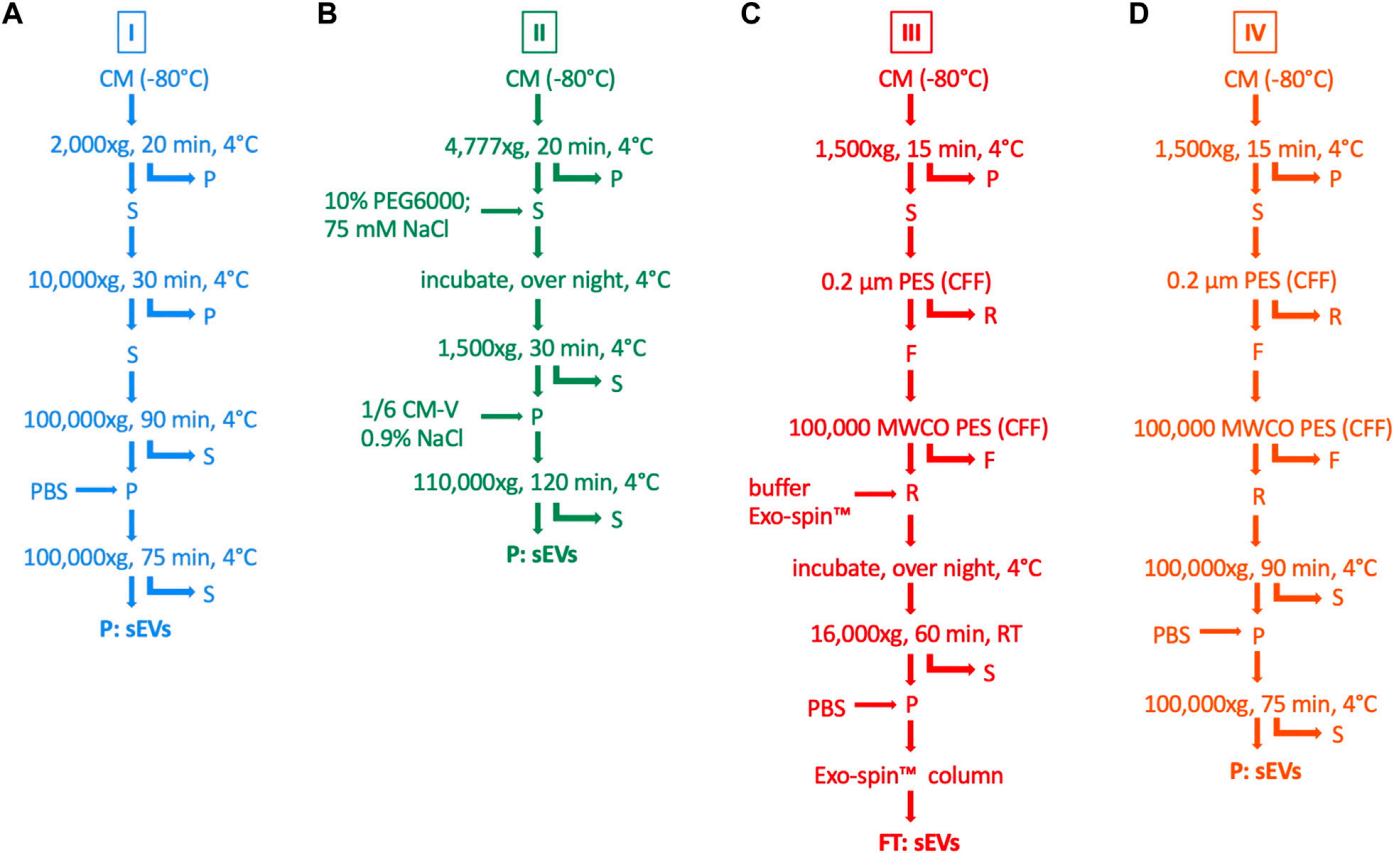
Methods for isolation of sEVs from MSC CM. sEVs were isolated from CM by four different isolation protocols (I—IV). **(A)** For protocol variant I (blue), CM was centrifuged at 2,000 × g and the pellet (P) was withdrawn. The supernatant (S) was centrifuged at 10,000 × g and the pellet was withdrawn again. These steps were performed for the removal of cellular debris and larger particles. The supernatant was centrifuged at 100,000 × g, the pellet was washed with PBS and centrifuged again in order to finally purify sEVs. **(B)** For protocol variant II (green), cellular debris and larger particles were removed by centrifugation with 4,777 × g followed by mixing the supernatant with 10% PEG6000 and 75 mM NaCl. After incubation overnight at 4°C, precipitated sEVs were centrifuged at 1,500 x g and resuspended in 0.9% NaCl, whereby the resuspension volume was defined as one-sixth of the initial volume of CM (CM-V). Finally, sEVs were enriched in the pellet after centrifugation with 110,000 × g. **(C)** For protocol variant III (red), centrifugation at 1,500 × g and CFF through a polyether sulfone (PES) filter membrane with a pore size of 0.2 µm was used for the elimination of cellular debris and larger particles in the pellet and retentate (R). Another CFF through a pore size of 100,000 MWCO was performed, thereby removing smaller particles in the filtrate (F) and reducing the volume for final sEV purification. The retentate was mixed with Exo-spin™ buffer, incubated over night at 4°C and centrifuged at 16,000 x g. The pellet was resuspended in PBS, and the sEVs were purified by SEC with Exo-spin™ columns. **(D)** For protocol variant IV (orange), steps for the removal of cellular debris and larger particles and volume reduction were the same as for protocol variant III. Finally, sEVs were enriched by UC at 100,000 x g for two times, including one washing step with PBS, as performed in protocol variant I.

sEVs were isolated from CM using four different isolation procedures, as summarized in Figure 1. For all isolation methods, CM was thawed at 4°C overnight. Protocol variant I was based on DC with a final sEV enrichment step by UC (Leblanc et al., 2017). First, cellular debris and larger particles were removed by centrifugation at 2,000 × g and 10,000 × g, respectively. Then, the supernatant was ultracentrifuged at 100,000 × g and the sEVs in the pellet were resuspended in PBS and washed by an additional UC step. Protocol variant II was a two-step protocol consisting of initial volume reduction of the starting material by precipitation with PEG followed by final sEV enrichment by UC (Ludwig et al., 2018). Cellular debris and larger particles were removed by centrifugation at 4,777 × g. The supernatant was mixed with 75 mM NaCl and 10% PEG6000 (Sigma-Aldrich Chemie GmbH) and incubated overnight. Precipitated sEVs were centrifuged at 1,500 × g and resuspended in 0.9% NaCl (Fresenius Kabi Deutschland GmbH, Bad Homburg, Germany). The resuspension volume was set to one-sixth of the original volume of the starting material, thereby leading to volume reduction for the final sEV enrichment step by UC at 110,000 × g. Protocol variant III was modified after Armacki et al. (2020) and included CFF for volume reduction of the starting material and final sEV purification by SEC. Briefly, cellular debris and larger particles were excluded by centrifugation at 1,500 × g followed by CFF using the Vivaflow 50 filter device (Sartorius Lab Instruments GmbH & Co., KG), with a pore size of 0.2 µm. Then, the suspension was filtered again using the Vivaflow 50 filter device (Sartorius Lab Instruments GmbH & Co., KG), with a pore size of 100,000 molecular weight cut-off (MWCO) in order to remove smaller particles and to reduce the volume for final sEV purification by the Exo-spin Exosome Purification kit (Cell Guidance Systems Ltd., Cambridge, United Kingdom). This procedure included an overnight incubation with Exo-spin™ buffer, centrifugation at 16,000 × g and SEC with Exo-spin™ columns. Protocol variant IV was a combined approach of protocol variant I and III, whereby CFF was utilized for initial volume reduction and final sEV enrichment was achieved by UC. The isolated sEVs were resuspended in 50–100 µL PBS (protocol variant I, III, and IV) or 0.9% NaCl (protocol variant II), respectively, and stored at −80°C.

#### Quantification of sEVs

Protein concentration of sEV suspensions was determined by bicinchoninic acid (BCA) assay using the Pierce™ BCA Protein Assay Kit (Thermo Fisher Scientific Inc.), as per manufacturer’s instructions. Absorbance at 562 nm was measured using a microplate reader POLARstar Omega (BMG LABTECH GmbH) with Reader Control and MARS Data Analysis software. Nanoparticle tracking analysis (NTA) was performed for the determination of particle concentration and size distribution of sEV suspensions. Analysis was carried out by NANOSIGHT NS300 (Malvern Instruments Limited, Malvern, United Kingdom) with NTA 3.4 Build software. For comparison of sEV isolation efficiency of methods I—IV, measured protein and particle concentrations were normalized to volumes of CM used for isolation. Protein and particle concentrations per cell were obtained by normalization to number of harvested cells. Purity of sEV suspensions was assessed by particles to protein ratio, as suggested by Webber and Clayton (2013).

#### Western blotting

The expression of different proteins by sEVs was verified by sodium dodecylsulfate polyacrylamide gel electrophoresis (SDS-PAGE) and western blotting using the Bolt™ Bis-Tris system with 2-(N-morpholino) ethanesulfonic acid (MES) buffer conditions (Thermo Fisher Scientific Inc.). Analyzed proteins were chosen according to Minimal Information for Studies of Extracellular Vesicles (MISEVs) 2018 criteria and included apolipoprotein A1 (ApoA1), CD63, CD81, flotillin-1 (Flot-1), and glucose-regulated protein 94 (GRP94) (Thery et al., 2018). β-actin was used as loading control. MSC cell lysate served as positive control for CD63, CD81, Flot-1, and GRP94. EV depl. PL was taken as positive control for ApoA1. PageRuler Plus Prestained Protein Ladder was used as a protein marker (Thermo Fisher Scientific Inc.). All steps were performed at RT unless stated otherwise. Briefly, proteins (5 µg for ApoA1, Flot-1, and GRP94 under reducing conditions; 10 µg for CD63 and CD81 under non-reducing conditions) were separated on a 12% polyacrylamide gel and subsequently blotted on a polyvinylidene fluoride (PVDF) membrane with a pore size of 0.2 µm. Reducing conditions were obtained by the addition of 10X Bolt™ Sample Reducing Agent (Thermo Fisher Scientific Inc.), containing 500 mM dithiothreitol (DTT). Membranes were blocked for 1 h in 5% milk (5% skimmed milk powder (J. M. Gabler-Saliter Milchwerk GmbH & Co., KG, Obergünzburg, Germany) in PBS with 0.1% Tween^®^20 (Sigma-Aldrich Chemie GmbH) (PBS-T)) and washed four times for 5 min in PBS-T. Primary antibodies were as follows: β-actin (clone AC-15; Sigma-Aldrich Chemie GmbH), 1:2,000 in 2% bovine serum albumin (BSA) (2% BSA (Sigma-Aldrich Chemie GmbH) in PBS-T), ApoA1 (clone EP1368Y; Abcam, Cambridge, United Kingdom), 1:1,000 or 1:2,000 in 5% milk, CD63 (clone MX-49.129.5; Santa Cruz Biotechnology, Inc., Dallas, United States), 1:1,000 in 5% milk, CD81 (clone JS-81; BD Biosciences), 1:1,000 in 5% BSA; Flot-1 (clone D2V7J; Cell Signaling Technology, Danvers, United States), 1:1,000 in 5% milk; GRP94 (polyclonal; Thermo Fisher Scientific Inc.), 1:1,000 in 5% milk. Incubation with primary antibodies was performed overnight at 4°C. Then, membranes were washed four times for 5 min in PBS-T and incubated with secondary antibodies for 1 h (Peroxidase AffiniPure Goat Anti-Mouse IgG, light chain specific (mouse-LC) for β-actin and CD63; Peroxidase AffiniPure F(ab')₂ Fragment Donkey Anti-Mouse IgG (H + L) (mouse) for β-actin, CD63 and CD81; Peroxidase AffiniPure F(ab')₂ Fragment Donkey Anti-Rabbit IgG (H + L) (rabbit) for ApoA1, Flot-1, and GRP94; all from Jackson ImmunoResearch Europe Ltd., Ely, United Kingdom). The membranes were washed four times for 5 min in PBS-T, and chemiluminescent signals were detected using SuperSignal™ West Pico PLUS Chemiluminescent Substrate (Thermo Fisher Scientific Inc.) and chemiluminescence detector Fusion FX (Vilber, Collégien, France) with Evolution-Capt software. Signals were quantified using Bio1D software.

#### Transmission electron microscopy (TEM)

sEVs were visualized by the negative stain technique. Briefly, 10 µL of sEV samples diluted with PBS were given on a glow discharged carbon-coated electron microscopy grid (Plano GmbH, Wetzlar, Germany) and incubated for 10 min at RT. Afterward, the grid was washed with three droplets of bi-distilled water prior to the addition of a drop of 2% uranyl acetate in water. Uranyl acetate was blotted with filter paper, and the samples were dried before they were observed in a transmission electron microscope JEM-1400Flash Electron Microscope (JEOL (Germany) GmbH, Freising, Germany) with iTEM software (Olympus Corporation, Tokyo, Japan) at 120 kV accelerating voltage and 60,000 times magnification.

#### Magnetic bead-based flow cytometric analysis

Surface antigen expression by sEVs was analyzed using the MACSPlex Exosome Kit, human (Miltenyi Biotec B.V. & Co., KG), as per the manufacturer’s instructions. Briefly, 5 µg sEVs were incubated with MACSPlex Exosome Capture Beads against 39 different surface antigen epitopes overnight at RT with agitation. Surface antigens included CD9, CD63, CD81, CD105, CD49e, stage-specific embryonic antigen-4 (SSEA-4), melanoma chondroitin sulfate proteoglycan (MCSP), CD146, CD44, CD29, CD62P, CD41b, CD42a, CD40, CD31, HLA ABC, CD45, HLA DPDQDR, CD24, CD69, CD19, CD4, CD3, CD8, CD56, CD2, CD1c, CD25, receptor tyrosine kinase-like orphan receptor 1 (ROR1), CD209, CD11c, CD86, CD326, CD133/1, CD142, CD20, CD14, REA control, and mIgG1 control. Bound sEVs were detected indirectly by allophyocyanin (APC)-coupled MACSPlex Exosome Detection Reagent directed against the tetraspanins CD9, CD63, and CD81 prior to flow cytometric analysis. Gating on single beads, median fluorescence intensity (MFI) of each capture bead population was measured using a FACSCelesta™ Cell Analyzer with BD FACSDiva™ software (BD Biosciences). Expression of each surface antigen was observed by subtracting MFI of the blank (buffer only) from MFI of the respective capture bead population and normalizing on mean MFI of CD9, CD63, and CD81, resulting in tetraspanin-normalized expression. Due to this indirect detection method, no information can be obtained on expression density, rather providing information about general positivity for each surface antigen. Hence, higher tetraspanin-normalized expression values mean more sEVs being positive for this surface antigen at all (Wiklander et al., 2018).

### Statistics

Statistical analysis was performed with GraphPad PRISM software version 9.3.1 (Graphpad Software Inc., San Diego, United States). For all experiments at least three independent experiments (N ≥ 3) were carried out, and data are presented as mean ± standard deviation (SD). Data were tested for normal distribution using the Shapiro–Wilk test and for homogenous variance using the Brown–Forsythe test. Significant differences between groups were investigated as follows. Comparison of two groups was carried out by unpaired t-test or Mann–Whitney test. For data of more than two groups, one-way analysis of variance (ANOVA) or Kruskal–Wallis test was performed. Dunnets and Dunn’s correction were applied for multiple testing. Data with inhomogenous variance were tested by one-way ANOVA with Welch correction and Dunnet T3 method for multiple testing. For proliferation assays, significant differences between groups and time points were assessed by two-way ANOVA with Geisser–Greenhouse correction, and Tukey correction was applied for multiple testing.

## Results

### Isolation of sEVs by different methods results in divergent quantity and purity

**FIGURE 2:**
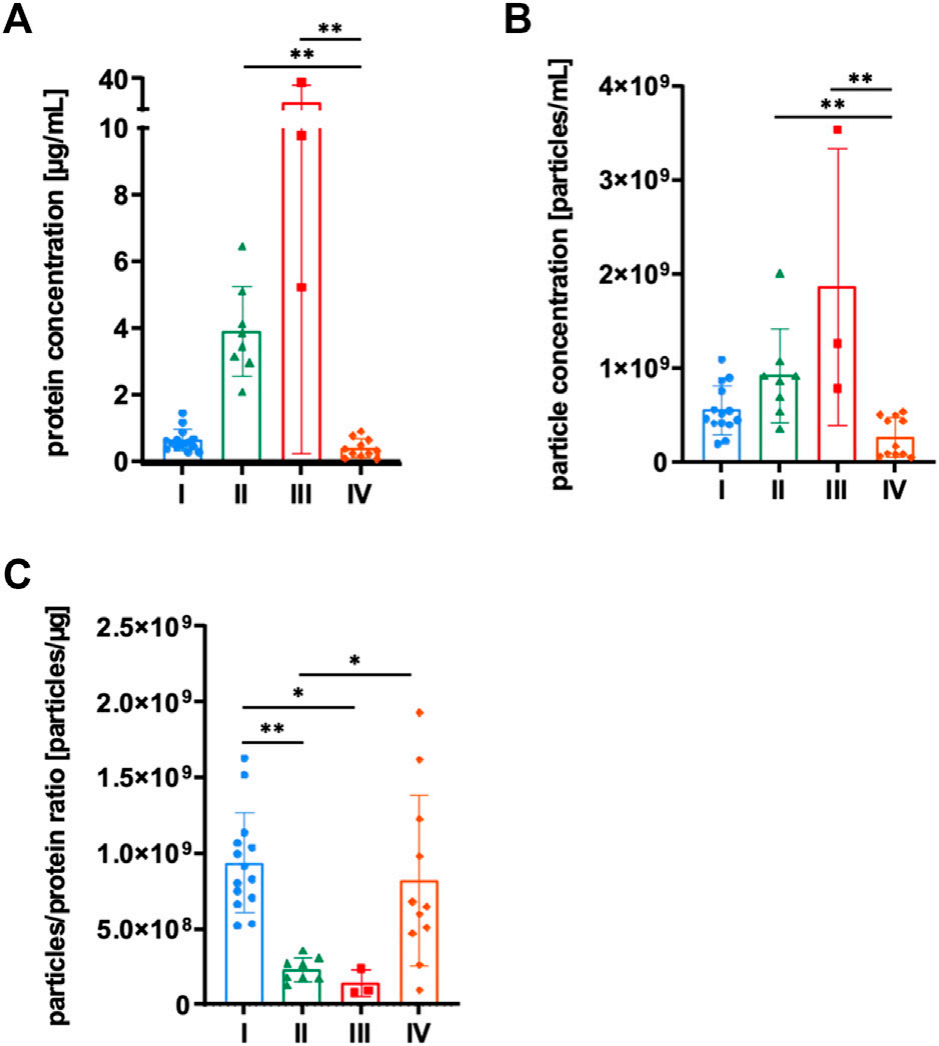
Quantification of sEVs. MSCs were grown in αMEM+8% PL, and sEVs were collected in CM for 24–48 h prior to isolation by methods I (blue), II (green), III (red), and IV (orange). **(A)** Protein concentration and **(B)** particle concentration of sEV preparations were determined by BCA assay and NTA, respectively. **(C)** Particles to protein ratio was calculated for information on purity of isolated sEVs. Data are presented as mean ± SD and N ≥ 3 independent experiments were performed. Statistically significant differences are depicted as follows: *: *p* < 0.05, **: *p* < 0.01.

MSCs were grown in αMEM+8% PL, and sEVs were collected in CM for 24–48 h prior to isolation by methods I—IV (Figure 1). Then, sEVs were quantified by BCA assay and NTA. Significantly higher protein and particle concentrations were achieved by methods II and III, respectively (Figures 2A, B). However, purity of aforementioned sEV preparations was significantly reduced as indicated by significantly lower particles to protein ratio (Figure 2C). No significant differences were observed between methods I and IV (Figure 2).

### Characteristics of sEVs purified with different isolation methods vary

**FIGURE 3:**
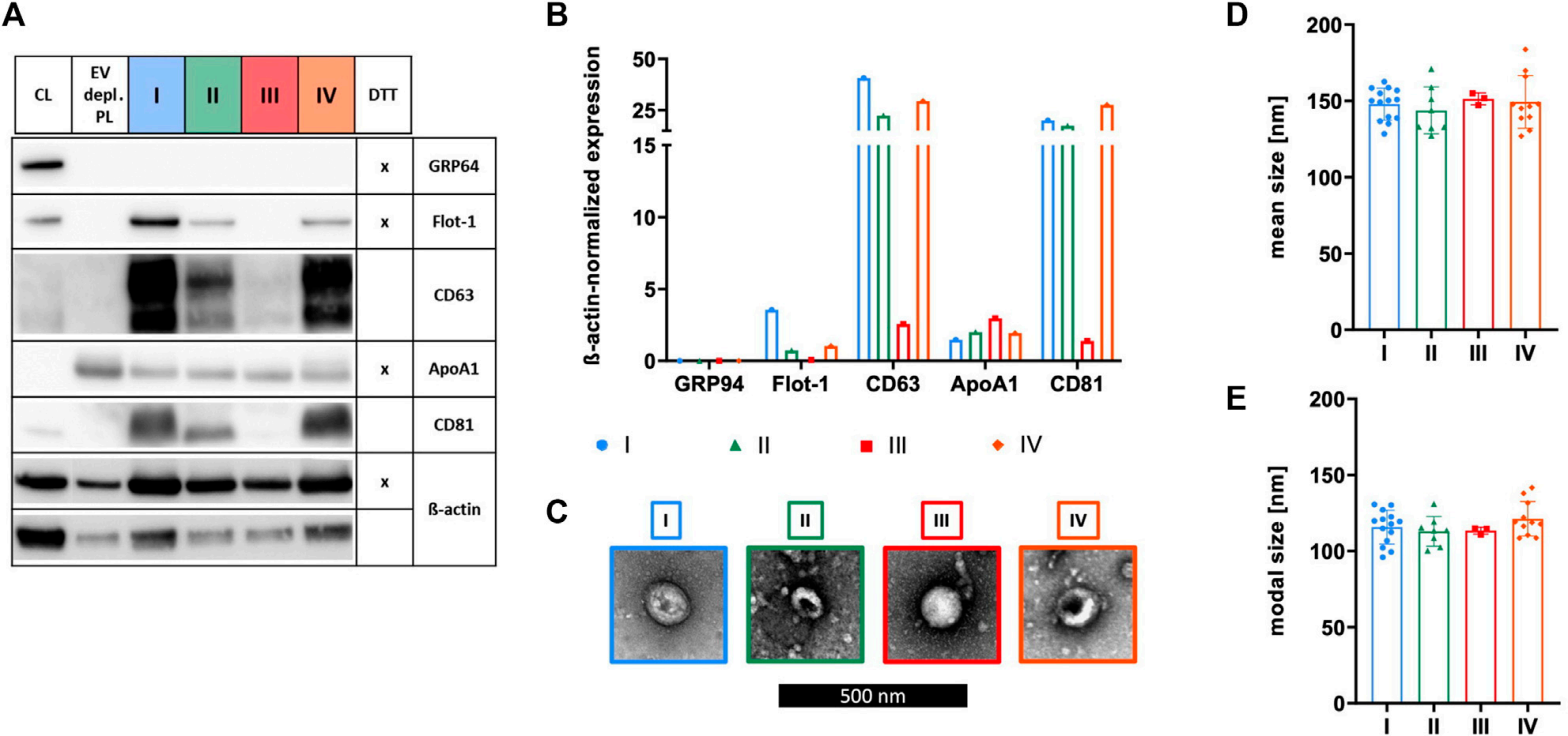
Characterization of sEVs. After isolation by methods I (blue), II (green), III (red), and IV (orange), sEVs were characterized. **(A)** Western blotting was performed in order to investigate the expression of proteins GRP94, Flot-1, CD63, ApoA1, and CD81. MSC cell lysate (CL) and EV depl. PL served as controls for primary antibodies, and ß-actin was used as loading control. Reducing conditions for indicated proteins were obtained by the addition of dithiothreitol (DTT). **(B)** Chemiluminescent signal intensities were quantified and normalized on ß-actin intensities, resulting in ß-actin-normalized expression of proteins. **(C)** sEVs were visualized by negative contrast staining using TEM with 60,000 times magnification. The black scale bar represents 500 nm. **(D)**+**(E)** Mean and modal size of sEVs was determined by NTA. Representative images are depicted for western blotting and TEM. Data are presented as mean ± SD, and N ≥ 3 independent experiments were performed for NTA analyses.

The identity of sEVs was proven by analyzing the existence of proteins according to the MISEV 2018 recommendation, including the presence of transmembrane/lipid-bound proteins (e.g., CD63 and CD81) or cytosolic proteins recovered in sEVs (e.g., Flot-1), and the absence of proteins of prominent contaminants co-isolated with sEVs (e.g., ApoA1) or proteins of intracellular compartments such as the Golgi apparatus (e.g., GRP94) (Thery et al., 2018). Expressions of Flot-1, CD63, and CD81 in addition to a lack of expression of GRP94 demonstrated isolation of sEVs by methods I, II, and IV. In contrast, method III failed in effectively isolating sEVs due to the absence of Flot-1 and CD81 and only marginal expression of CD63. Presence of co-isolated proteins for all isolation methods was indicated by ApoA1, being the lowest for sEVs isolated by method I (Figures 3A, B). TEM verified the existence of sEVs by microscopic images displaying enclosed particles in size range between approximately 100–200 nm (Figure 3C). More precise size ranges of sEVs were determined by NTA, where no significant differences in size distribution of sEVs isolated by methods I—IV were observed, as shown by similar mean and modal particle sizes (Figures 3D, E).

### Surface antigen expression of sEVs depends on the isolation method

**FIGURE 4:**
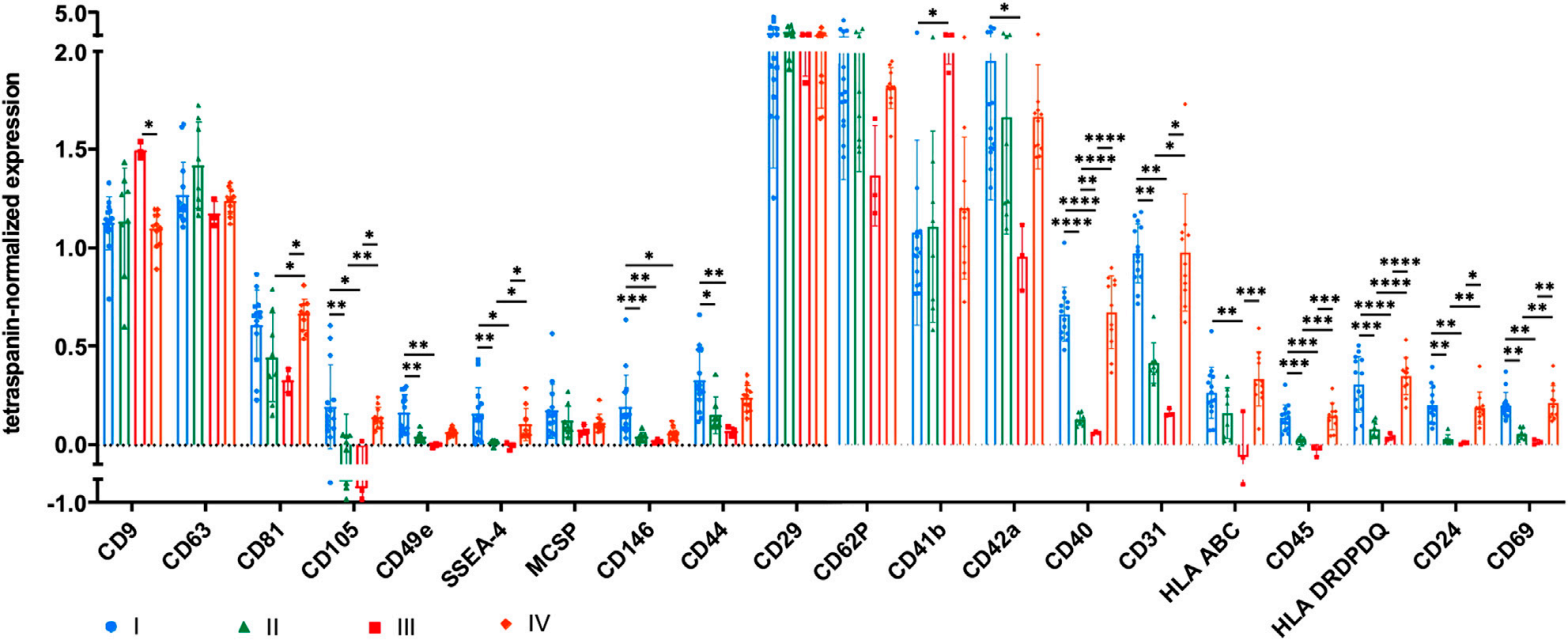
Tetraspanin-normalized surface antigen expression by sEVs. Surface antigen expression of sEVs isolated by methods I (blue), II (green), III (red) and IV (orange) was analyzed using MACSPlex technology. sEVs were bound by capture beads with epitopes against each analyzed surface antigen and detected indirectly by an APC-coupled detection reagent directed against the tetraspanins CD9, CD63, and CD81. Due to the indirect detection, fluorescence intensity of each surface antigen was normalized on mean fluorescence intensity of CD9, CD63, and CD81, resulting in tetraspanin-normalized expression. Data are presented as mean ± SD, and N ≥ 3 independent experiments were performed. Statistically significant differences are depicted as follows: *: *p* < 0.05, **: *p* < 0.01, ***: *p* < 0.001, ****: *p* < 0.0001.

The expression of several surface antigens by sEVs was analyzed using MACSPlex technology. While significant differences between sEVs isolated by methods I and IV were only observed for CD146, numerous significant differences were obtained when sEVs were purified with methods II and III (CD9, CD81, CD105, CD49e, SSEA-4, CD146, CD44, CD41b, CD42a, CD40, CD31, HLA ABC, CD45, HLA DRDPDQ, CD24, and CD69; Figure 4). Tetraspanin-normalized expression of surface markers tended to be the highest in sEVs isolated by protocol I (CD105, CD49e, SSEA-4, CD146, CD44, and CD42a) and protocol IV (CD81, HLA ABC, and HLA DRDPDQ) and the lowest in sEVs isolated by protocol III (CD81, CD105, CD49e, CD146, CD44, CD42a, CD40, CD31, HLA ABC, CD45, HLA DRDPDQ, and CD69). In contrast, the tetraspanin-normalized expressions of CD9 and CD41b were the highest for sEVs purified by method III. Analysis of additional surface antigens analyzed is presented in Supplementary Figure S1.

### Expansion of MSCs in EV-depleted PL does not negatively affect MSC characteristics

**FIGURE 5:**
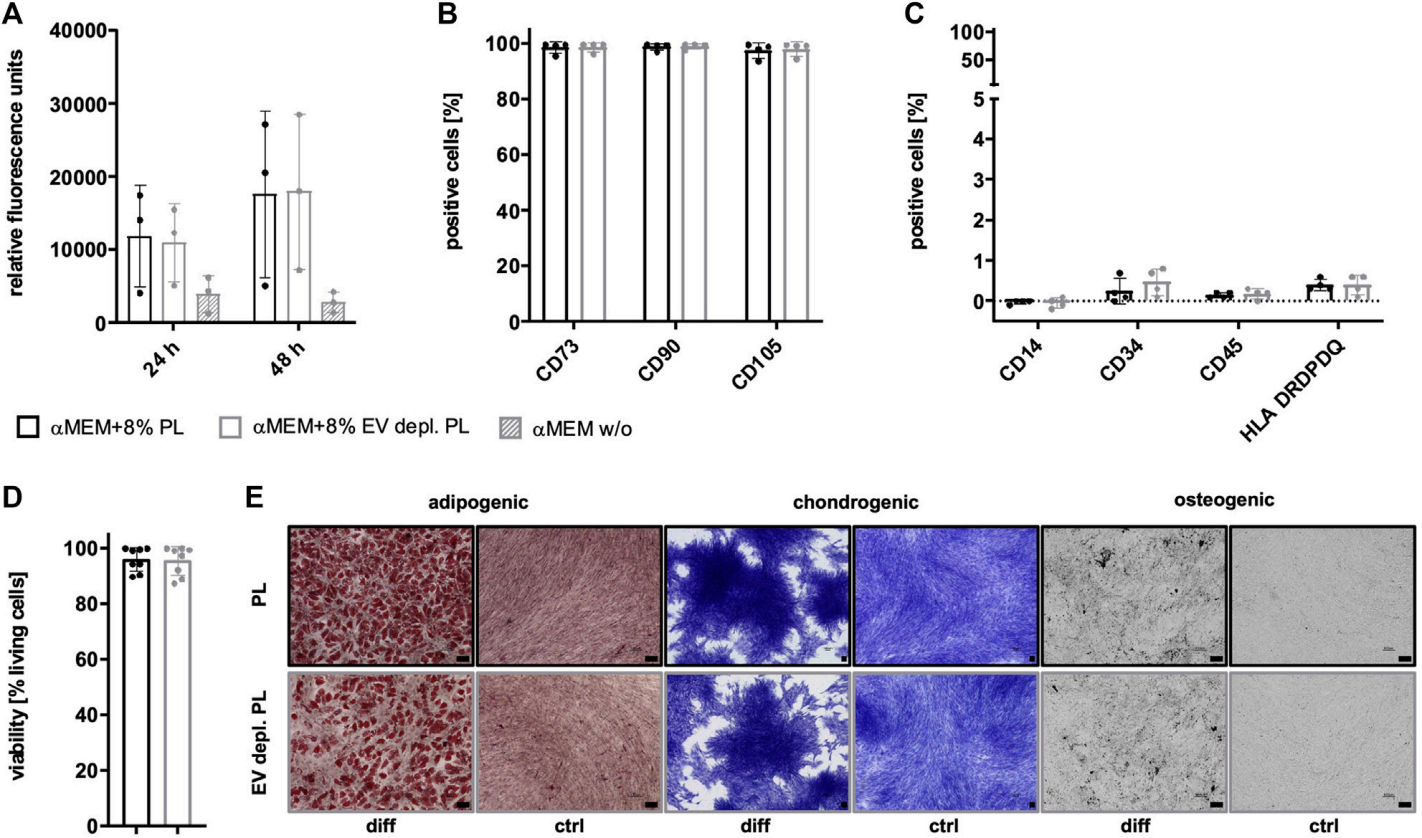
Characteristics of MSCs after expansion in different media. **(A)** MSCs were expanded in αMEM+8% PL (black), αMEM+8% EV depl. PL (gray), and αMEM without supplement (αMEM w/o; gray with stripes) in a 96-well plate (200 cells per well) for 24 h and 48 h, respectively. Triplicates (*n* = 3) were performed for each. The cells were stained with the CyQUANT Cell Proliferation Assays kit and, proliferation of cells was determined by fluorescence intensities. **(B–E)** MSCs were seeded at 2,000 cells/cm^2^ in αMEM+8% PL and αMEM+8% EV depl. PL and expanded for P1 or P2. Expression of identity markers CD73, CD90, and CD105 **(B)** and lack of expression of purity markers CD14, CD34, CD45, and HLA DRDPDQ **(C)** was analyzed by flow cytometry. **(D)** Viability of cells was determined by trypan blue staining. **(E)** Harvested MSCs of αMEM+8% PL and αMEM+8% EV depl. PL cultures were differentiated into cells of adipogenic, chondrogenic, and osteogenic lineages. For this, the cells were grown in specific differentiation media (diff) or in control media αMEM+20% FBS (ctrl) lacking differentiation-inducing substances. Adipogenic differentiation was verified by Oil Red O and hematoxylin staining, chondrogenic differentiation was demonstrated by methylene blue staining and osteogenic differentiation was proven by activity of alkaline phosphatase using BCIP/NBT substrate. Pictures were taken with 4 times (chondrogenic) and 10 times magnification (adipogenic and osteogenic), respectively. Black scale bars indicate 100 µm. Data are presented as mean ± SD, and N ≥ 3 **(A–D)** or N = 2 **(E)** independent experiments were performed. Representative images are depicted for differentiation assays.

Due to the existence of PL-derived sEVs in αMEM+8% PL, different collection strategies were exerted during MSC expansion in order to enrich for and verify the presence of MSC-derived sEVs. In addition to collecting sEVs during a starvation period in basal media αMEM (αMEM w/o) lacking any additional supplement, EV depletion of PL was examined as a suitable strategy for the removal of contaminating sEVs while still containing other growth promoting supplements. As a first step, proliferation of MSC in αMEM+8% PL, αMEM+8% EV depl. PL and αMEM w/o was compared for 24 h and 48 h, respectively, to check for effects on MSC proliferation. Due to a highly decreased proliferation rate of MSCs grown in αMEM w/o, this medium was excluded from further analyses (Figure 5A). No negative impact of expansion in αMEM+8% EV depl. PL was observed on the expression of identity and purity markers (Figures 5B, C), viability of cells (Figure 5D), and tri-lineage differentiation potential (Figure 5E) as compared to cells grown in αMEM+8% PL.

### Different collection strategies allow for the generation of varying sEV compositions

**FIGURE 6:**
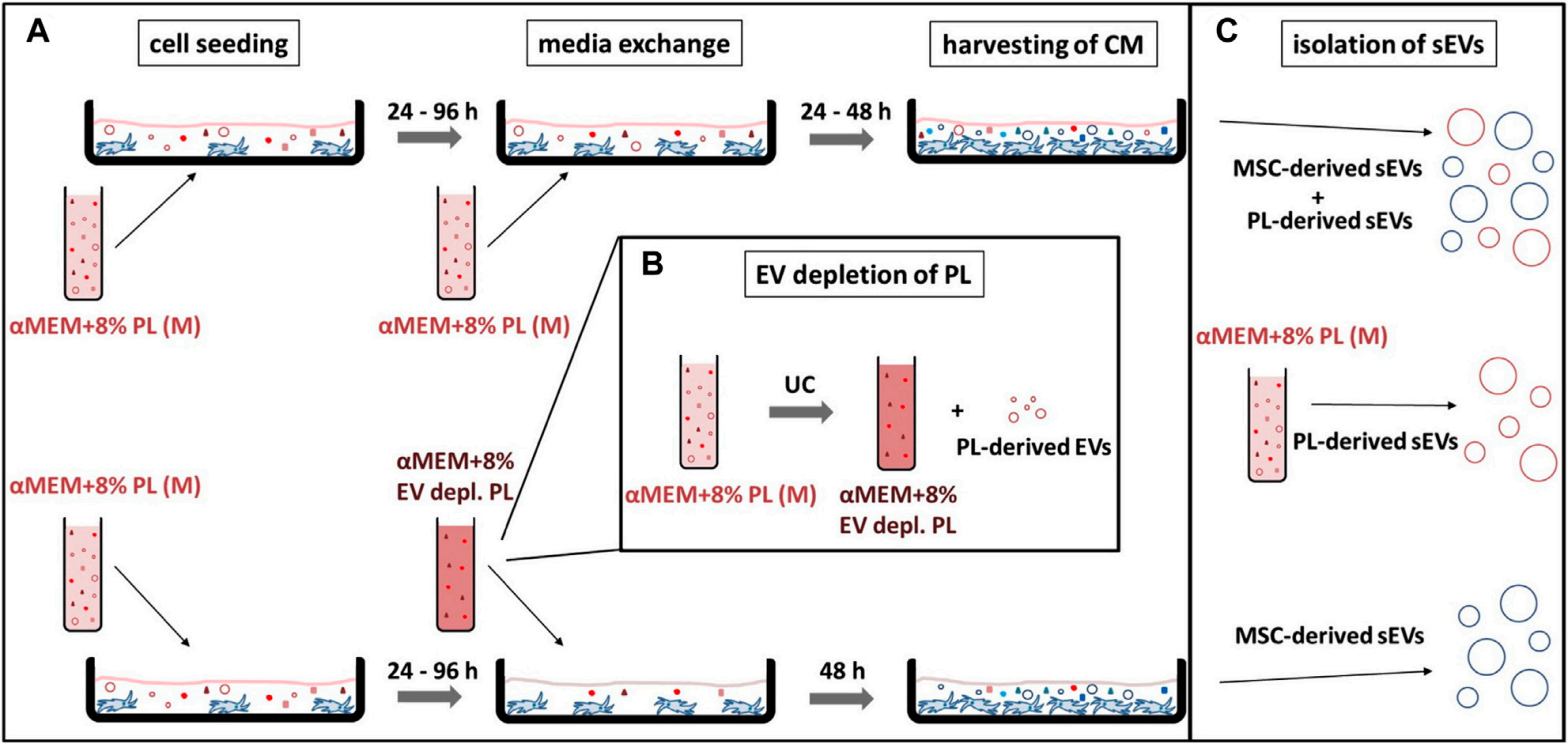
Workflow of different sEV collection strategies. **(A)** MSCs were seeded at 2,000–4,000 cells/cm^2^ in the media αMEM+8% PL (M) and expanded for P1 or P2. After 24–96 h media was exchanged completely for the collection of sEVs in CM. In addition to αMEM+8% PL, αMEM+8% EV depl. PL was chosen as collection media for enrichment of MSC-derived sEVs. In order to exclude an impact of batch-to-batch variation of PL, the same starting batches of PL (from the same donors) were used for the experiments shown in the upper and lower row, which just differed whether or not EV depletion has been performed. Collection time was set to 24 or 48 h for αMEM+8% PL cultures and to 48 h for αMEM+8% EV depl. PL cultures. CM was harvested and stored at −80°C until isolation of sEVs. **(B)** αMEM+8% EV depl. PL was manufactured by UC of PL for 3 h at 120,000 × g at 4°C, whereby the pellet containing the PL-derived EV was withdrawn. **(C)** sEVs isolated from CM collected with αMEM+8% PL included MSC-derived and PL-derived sEVs, whereas sEVs collected with αMEM+8% EV depl. PL solely included MSC-derived sEVs. PL-derived sEVs were isolated from the media αMEM+8% PL to confirm the hypothesis.

In addition to collection media, elongation of collection time could also be beneficial for enriching MSC-derived sEVs. Therefore, collection for 48 h in αMEM+8% EV depl. PL was investigated as strategy for exclusively generating MSC-derived sEVs. In order to confirm the effectiveness of this strategy, sEVs were isolated from the media αMEM+8% PL and MSC CM collected for 24 h or 48 h in αMEM+8% PL (Figure 6). After having tested different sEV isolation procedures during the first part of the study, only method IV was considered as suitable for sEV isolation from MSC CM besides gold standard method I due to consistent characteristics and purity.

### Expansion of MSCs in αMEM+8% EV depl. PL leads to enrichment of MSC-derived sEVs

**FIGURE 7:**
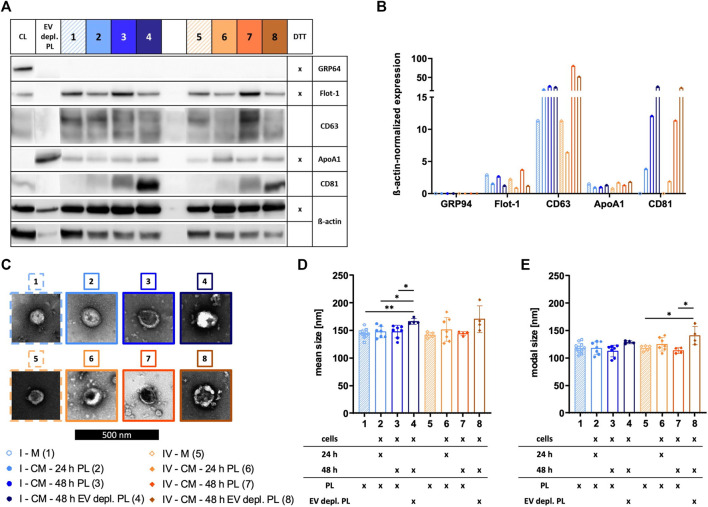
Characterization of sEVs collected with different strategies. sEVs were isolated from αMEM+8% PL (M) (1 and 5; with stripes) in order to verify the presence of PL-derived sEVs in the media. MSCs were grown in αMEM+8% PL and αMEM+8% EV depl. PL. sEVs were collected for 24 h (2 and 6) and 48 h (3 and 7) for αMEM+8% PL cultures and for 48 h for αMEM+8% EV depl. PL cultures (4 and 8). Methods I (blue scheme) and IV (orange scheme) were used for sEV isolation from M and CM. **(A)** Western blotting was performed in order to investigate the expression of proteins GRP94, Flot-1, CD63, ApoA1, and CD81. MSC cell lysate (CL) and EV depl. PL served as controls for primary antibodies, and ß-actin was used as loading control. Reducing conditions for indicated proteins were obtained by the addition of dithiothreitol (DTT). **(B)** Chemiluminescent signal intensities were quantified and normalized on ß-actin intensities, resulting in ß-actin-normalized expression of proteins. **(C)** sEVs were visualized by negative contrast staining using TEM with 60,000 times magnification. The black scale bar represents 500 nm. **(D)** + **(E)** Mean and modal size of sEVs was determined by NTA. Representative images are depicted for western blotting and TEM. Data are presented as mean ± SD, and N ≥ 4 independent experiments were performed for NTA analyses. Statistically significant differences are depicted as follows: *: *p* < 0.05, **: *p* < 0.01.

The identity of sEVs was demonstrated for sEV preparations from the media αMEM+8% PL and MSC CM collected for 24 h or 48 h in αMEM+8% PL or αMEM+8% EV depl. PL, as shown by the protein expression of Flot-1 and CD63 and lack of expression of GRP94. Being absent in sEVs from the media, CD81 increased with collection time and showed the highest expression for sEVs from EV depl. PL conditions, providing evidence for effective enrichment of MSC-derived sEVs. Similar amounts of co-isolated proteins were found in all sEV preparations indicated by the presence of ApoA1 (Figures 7A, B). TEM further verified sEV identity with microscopic pictures, displaying membrane-surrounded particles for all preparations (Figure 7C). Regarding size distribution of sEVs determined by NTA, the mean and modal particle sizes of sEVs from αMEM+8% EV depl. PL cultures (groups 4 and 8 in Figures 7D, E) were higher than those of both, sEVs from media (groups 1 and 5 in Figures 7D, E) and sEVs from αMEM+8% PL cultures (groups 2, 3, 6, and 7 in Figures 7D, E). This provided evidence for differences in size for PL- and MSC-derived sEVs being larger for the latter.

### sEVs collected by different strategies differ in surface antigen expression

**FIGURE 8:**
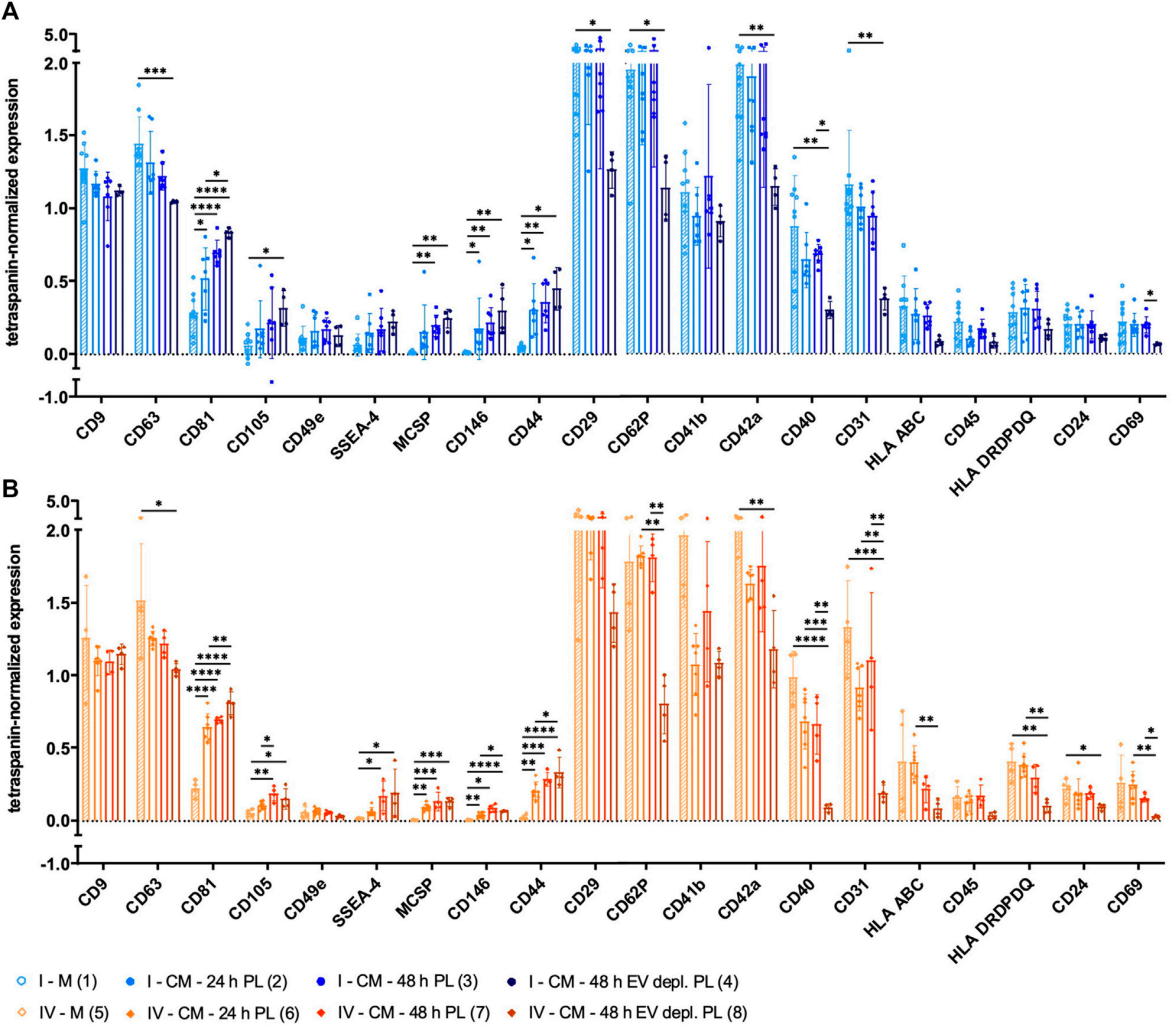
Tetraspanin-normalized surface antigen expression by sEVs collected with different strategies. sEVs were isolated from the media αMEM+8% PL (M) (1 and 5; with stripes) and MSC CM, where sEVs were collected for 24 h (2 and 6) and 48 h (3 and 7) for αMEM+8% PL cultures and for 48 h for αMEM+8% EV depl. PL cultures (4 and 8). Methods I (blue scheme) **(A)** and IV (orange scheme) **(B)** were used for sEV isolation from M and CM. The expression of several surface antigens by sEVs was analyzed using MACSPlex technology. sEVs were bound by capture beads with epitopes against each analyzed surface antigen and detected indirectly by an APC-coupled detection reagent directed against the tetraspanins CD9, CD63, and CD81. Due to the indirect detection, fluorescence intensity of each surface antigen was normalized on mean fluorescence intensity of CD9, CD63, and CD81, resulting in tetraspanin-normalized expression. Data are presented as mean ± SD, and N ≥ 4 independent experiments were performed. Statistically significant differences are depicted as follows: *: *p* < 0.05, **: *p* < 0.01, ***: *p* < 0.001, ****: *p* < 0.0001.

Surface antigen expression of sEVs derived from media αMEM+8% PL and MSC CM collected with different strategies was analyzed. Positivity for several MSC surface antigens was significantly increased for sEVs isolated from CM, and the highest positivity was obtained for those from αMEM+8% EV depl. PL cultures. These included, e.g., CD81, CD105, SSEA-4, MCSP, CD146, and CD44 (Figures 8A, B). In contrast, significantly less sEVs from αMEM+8% EV depl. PL cultures were positive for the surface antigens CD63, CD29, CD62P, CD42a, CD40, CD31, HLA DRDPDQ, and CD24 in comparison to those from media (Figures 8A, B). Significant differences were also observed between CM sEVs, both collected for 48 h but with different supplements. In comparison to sEVs from αMEM+8% EV depl. PL cultures, significantly more sEVs from αMEM+8% PL cultures were positive for CD62P, CD40, CD31, and CD69 (Figures 8A, B). Analysis of additional surface antigens is presented in Supplementary Figure S2. Diversity in surface antigen expression patterns for sEVs from media and CM was equally indicative for the presence of MSC-derived sEVs, increasing with collection time and EV depl. PL culture condition.

### Hollow fiber bioreactor-based MSC expansion does not negatively affect MSC characteristics

**FIGURE 9:**
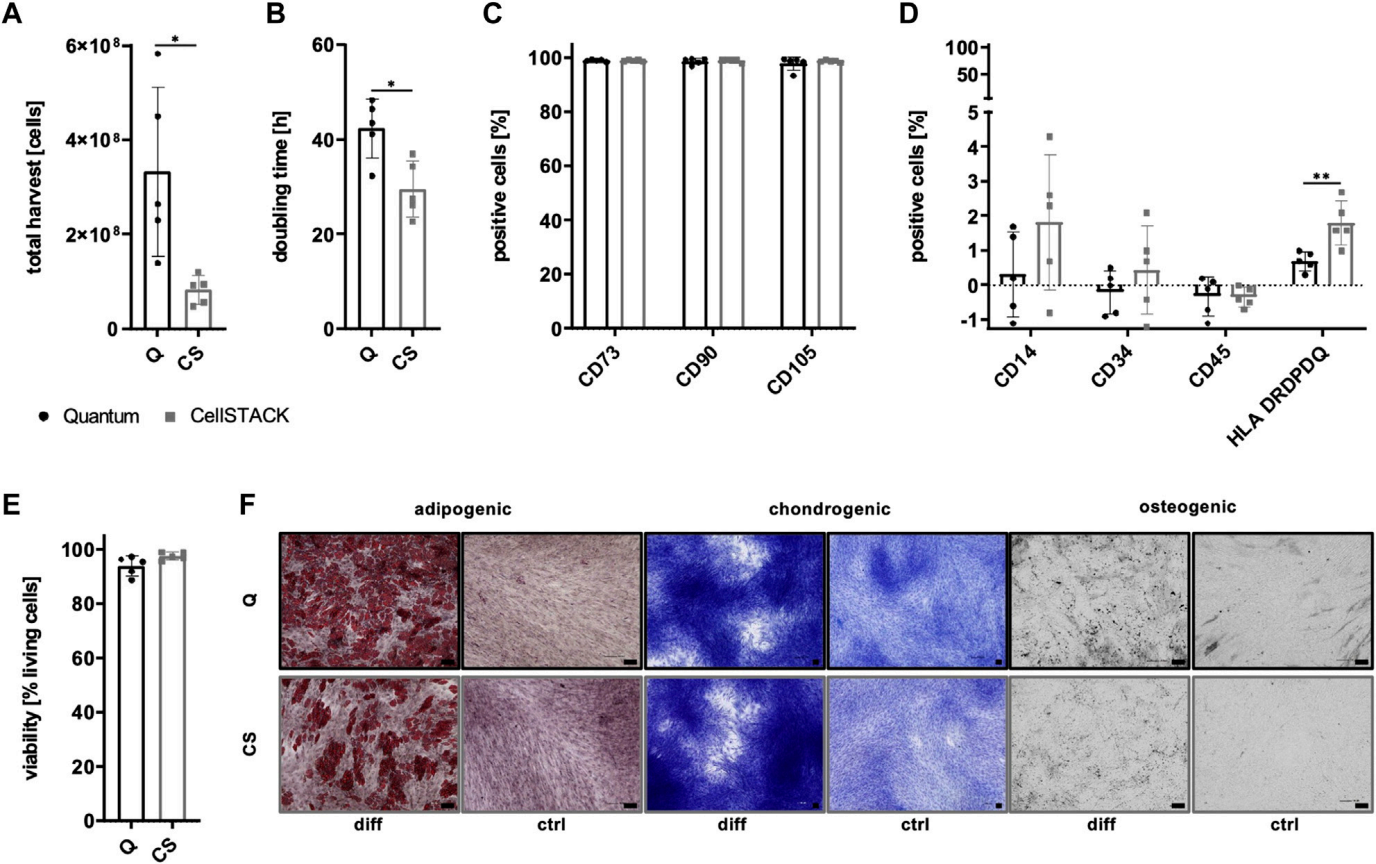
Expansion parameters and characteristics of MSCs after Quantum- and CellSTACK-based culture. MSCs were grown in αMEM+8% PL in a CP-coated Quantum hollow fiber bioreactor (Q; black) or in conventional cell culture in a CellSTACK (CS; gray) for P1. The expansion parameters total harvest **(A)** and doubling time **(B)** were determined after the harvesting of cells. The expression of identity markers CD73, CD90, and CD105 **(C)** and purity markers CD14, CD34, CD45, and HLA DRDPDQ **(D)** were analyzed by flow cytometry. **(E)** Viability of cells was determined by trypan blue staining. **(F)** Harvested MSCs were differentiated into cells of adipogenic, chondrogenic, and osteogenic lineages. For this, the cells were grown in specific differentiation media (diff) or in control media αMEM+20% FBS (ctrl) lacking differentiation-inducing substances. Adipogenic differentiation was verified by Oil Red O and hematoxylin staining, chondrogenic differentiation was demonstrated by methylene blue staining and osteogenic differentiation was proven by activity of alkaline phosphatase using BCIP/NBT substrate. Pictures were taken with 4 times (chondrogenic) and 10 times magnification (adipogenic and osteogenic), respectively. The black scale bars indicate 100 µm. Data are presented as mean ± SD, and N ≥ 4 independent experiments were performed. Representative images are depicted for differentiation assays. Statistically significant differences are depicted as follows: *: *p* < 0.05, **: *p* < 0.01.

In order to allow large-scale manufacturing of sEVs, a hollow fiber bioreactor-based expansion process was implemented. As a first step, important expansion parameters and cell characteristics were compared for MSCs grown in the CP-coated Quantum system and in conventional CellSTACK-based culture. Significantly, more cells could be harvested for the Quantum system with mean harvested cell numbers of more than 300 million cells showing its potential for large-scale expansion (Figure 9A), although cells grew significantly slower than that of conventional cell culture (Figure 9B). Significant differences in surface antigen expression of important identity (CD73, CD90, and CD105; Figure 9C) and purity markers (CD14, CD34, CD45; Figure 9D) were not observed between MSCs of both expansion systems except for significantly lower expression of HLA DRDPDQ for Quantum-derived MSCs (Figure 9D). Viability of cells was not significantly altered by the bioreactor-based expansion process (Figure 9E), and the differentiation capacity of MSCs toward cells of the adipogenic, chondrogenic, and osteogenic lineages could be maintained (Figure 9F).

### Hollow fiber bioreactor allows for the generation of sEVs with consistent properties

**FIGURE 10:**
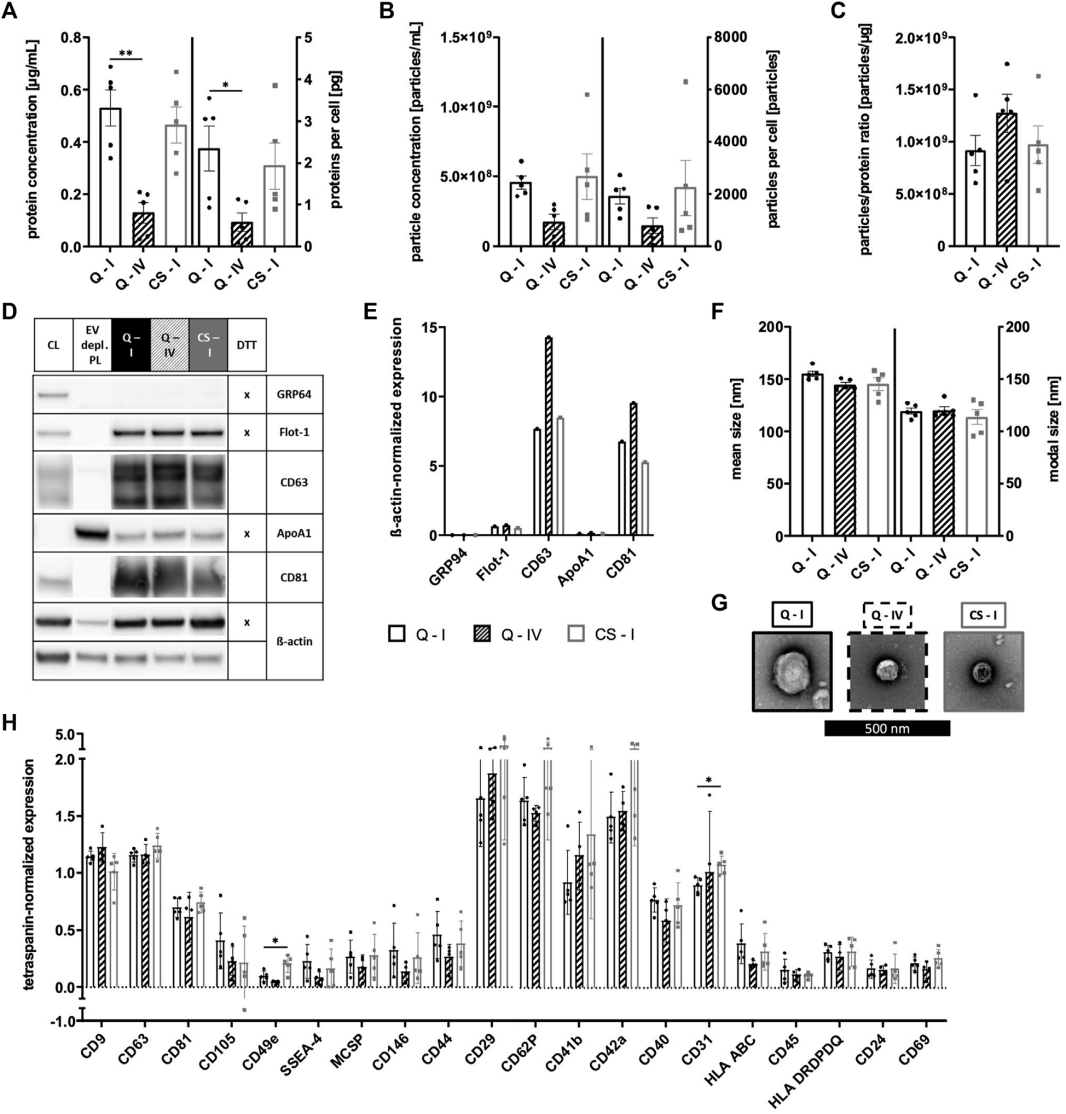
Quantification and characterization of sEVs from Quantum- and CellSTACK-based MSC expansion. sEVs were collected in CM for Quantum- (Q) and CellSTACK-based expansion (CS), respectively. sEVs were isolated from CM by method I for both expansion systems (Q—I; black and CS—I; gray) in addition to method IV for Quantum-derived CM (Q—IV; black with stripes). **(A)** Protein concentration of sEVs and proteins per cell were quantified by BCA assay. **(B)** Particle concentration and particles per cell were analyzed by NTA. **(C)** Particles to protein ratio was calculated for information on purity of isolated sEVs. **(D)** Western blotting was performed in order to investigate the expression of proteins GRP94, Flot-1, CD63, ApoA1, and CD81. MSC cell lysate (CL) and EV depl. PL served as controls for primary antibodies, and ß-actin was used as loading control. Reducing conditions for indicated proteins were obtained by the addition of dithiothreitol (DTT). **(E)** Chemiluminescent signal intensities were quantified and normalized on ß-actin intensities, resulting in ß-actin-normalized expression of proteins. **(F)** Mean and modal size of sEVs was determined by NTA. **(G)** sEVs were visualized by negative contrast staining using TEM with 60,000 times magnification. The black scale bar represents 500 nm. **(H)** The expression of several surface antigens by sEVs was analyzed using MACSPlex technology. sEVs were bound by capture beads with epitopes against each analyzed surface antigen and detected indirectly by an APC-coupled detection reagent directed against the tetraspanins CD9, CD63, and CD81. Due to the indirect detection, fluorescence intensity of each surface antigen was normalized on mean fluorescence intensity of CD9, CD63, and CD81, resulting in tetraspanin-normalized expression. Representative images are depicted for western blotting and TEM. Data are presented as mean ± SD, and N ≥ 4 independent experiments were performed for BCA assay, NTA and MACSPlex analyses. Statistically significant differences are depicted as follows: *: *p* < 0.05, **: *p* < 0.01.

sEVs were collected in CM for 16–19 h for the Quantum system and for 24–48 h for CellSTACK-based expansion. As the presence of MSC-derived sEVs in CM could be proven during previous parts of this study, αMEM+8% PL was used as growth media for both expansion systems in order to exclude changes of sEV composition by alteration of culture conditions. Isolation of sEVs by method I was carried out for both expansion systems, whereas method IV was only used for Quantum-derived CM to check its potential for large-scale applicability. No significant differences in quantity were observed between sEVs isolated from the two expansion systems by method I as protein and particle concentrations as well as proteins and particles per cell were in similar ranges (Figures 10A, B). Lower protein and particle concentrations were obtained for isolation by method IV (Figures 10A, B); however, elevated particles to protein ratio indicated higher purity of respective sEVs (Figure 10C). sEVs from both expansion systems expressed proteins Flot-1, CD63, and CD81, where the highest levels of tetraspanins CD63 and CD81 were observed for Quantum-derived sEVs isolated by method IV (Figures 10D, E). While GRP94 was absent in all sEV preparations, co-isolated proteins were found at similar levels as indicated by the expression of ApoA1 (Figures 10D, E). Analyses of sEV size showed particles in size range between 100–200 nm with no significant differences between the expansion systems and isolation methods (Figure 10F). TEM confirmed the presence of sEVs in all preparations (Figure 10G). Similar surface antigen expression patterns were observed for all sEV preparations with some markers being more frequently positive for sEVs of CellSTACK-derived expansion (e.g., CD49e, CD29, CD62P, CD41b, CD42a, and CD31; Figure 10H). Analysis of additional surface antigens is presented in Supplementary Figure S3.

## Discussion

Implementation of standardized large-scale applicable procedures for the manufacturing of sEVs is of high relevance in order to overcome barriers currently impeding translation of laboratory-scale protocols toward clinical applications. This not only includes lacking standardization of culture conditions during sEV production but also of sEV isolation methods. In addition, changing from flask-based expansion processes to a (semi-) automated expansion in bioreactors is still hampered and thus prevents efficient upscaling strategies (Paolini et al., 2022).

For this reason, we investigated possible improvements at various levels: (i) modification of sEV isolation protocols and their impact on efficacy of isolation and the sEV characteristics obtained thereby, (ii) analysis of the impact of culture characteristics (e.g., duration and supplements) on sEV output, and (iii) upscaling of the process toward a bioreactor-based large-scale expansion and generation of CM as a starting material for sEV preparation. Based on our results, we propose a hollow fiber bioreactor-based process for the generation of BM MSC-derived sEVs that, in combination with isolation by CFF and UC, allows for large-scale production of sEVs. Optionally, EV-depleted supplements (e.g., EV depl. PL) could be used in order to enrich for MSC-derived sEVs and to avoid supplement-derived sEVs in the final sEV preparation.

DC with a final enrichment of sEVs by UC is still considered as gold standard for the isolation of sEVs. However, due to its limited applicability for large-scale purification of sEVs from large volumes of starting material, various approaches combining different isolation methods became increasingly popular (Gardiner et al., 2016). CFF or precipitation with PEG can be applied for volume reduction of starting material and, besides UC, SEC can be used for final enrichment of sEVs. In this study, different combinations of isolation methods were evaluated. Only a combination of CFF with UC (method IV) was considered as suitable for the isolation of MSC-derived sEVs in a large-scale setting. It combines the advantages of CFF-based volume reduction with an efficiency of a UC-derived sEV preparation in the subsequent step. sEVs obtained by this method showed similar characteristics as those purified with the gold standard method DC with UC (method I). This combination was also proposed by Rohde et al. for a good manufacturing practice (GMP)-compliant manufacturing process of MSC-derived sEVs (Rohde et al., 2019) and has been recently implemented for a first-in-human intracochlear application (Warnecke et al., 2021). Both, combination of PEG with UC (method II) and CFF with SEC (method III) resulted in altered sEV characteristics, as shown by different surface antigen expression patterns and in sEV preparations with low purity indicated by a lower particles to protein ratio (Webber and Clayton, 2013). Purity concerns already arose for PEG precipitation-based sEV isolation in other studies (Van Deun et al., 2014; Lobb et al., 2015). In contrast, SEC commonly led to preparations with high purity (Baranyai et al., 2015; Nordin et al., 2015). One possible explanation for the divergent results could be the intermediate step of incubation with Exo-spin™ buffer prior to final isolation of sEVs by SEC in this study, whereas an intermediate precipitation was not performed by Baranyai et al. and Nordin et al. In accordance with our results, Lobb et al. also found high amount of co-isolated albumin after sEV isolation with the Exo-spin™ system (Lobb et al., 2015). General critical considerations were also made by Witwer et al. in the context of using commercial kits not stating the exact composition of ingredients for GMP-grade sEV production (Witwer et al., 2019).

Since Torreggiani et al. showed the presence of sEVs in PL (Torreggiani et al., 2014), we wanted to prove the co-existence of MSC-derived sEVs in CM and evaluate strategies for enrichment of the latter. For this purpose, sEVs were isolated from the media αMEM+8% PL in addition to CM collected with different strategies. These included collection for 24 h or 48 h and collection during a 48 h period, in which EV depl. PL was used. As the identity of sEVs could be shown for all approaches, the production of MSC-derived sEVs could especially be verified by the EV depl. PL approach. PL- and MSC-derived sEVs significantly differed in their surface antigen expression pattern with most pronounced disparities for surface antigens CD81, CD105, SSEA-4, MCSP, CD146, CD44 (all more frequently expressed by MSC-derived sEVs), CD63, CD29, CD62P, CD42a, CD40, CD31, HLA DRDPDQ, and CD24 (all more frequently expressed by PL-derived sEVs). In addition, differences between sEVs from αMEM+8% PL and αMEM+8% EV depl. PL cultures were observed for surface antigen expression of known platelet markers CD62P, CD40, CD31, and CD69 (Stenberg et al., 1985; Newman et al., 1990; Testi et al., 1990; Inwald et al., 2003). These observations could be partially explained in that sEVs of EV depl. PL cultures totally lacked PL‐derived sEVs, thus including less sEVs positive for typical platelet markers. Although contaminating sEVs might potentiate therapeutic action in some cases, counteraction of proper functionality of MSC-derived sEVs could also be observed (Witwer et al., 2019). EV depletion of cell culture supplements is commonly applied in order to get rid of contaminating sEVs. As alteration of culture conditions has been shown to affect sEV characteristics (Li et al., 2015; Haraszti et al., 2019), critical considerations were made about this strategy. Although EV depl. PL showed no negative impact on important MSC characteristics in this study (e.g., expression of identity and purity markers, tri-lineage differentiation potential, and viability), changes in composition of MSC-derived sEVs as a consequence of cellular stress by altered culture conditions cannot be excluded (Wiest and Zubair, 2020; Almeria et al., 2022). Gobin et al. proposed CD40 as an important surface antigen responsible for immunomodulatory capacities of MSC-derived sEVs (Gobin et al., 2021). Since the latter is only marginally expressed by sEVs from αMEM+8% EV depl. PL cultures, this may in general raise questions about the efficacy of sEVs generated with deprivation strategies. Hence, changes of culture conditions always need to be critically evaluated depending on specific purposes. Our preference is the use of PL, which has not been EV depleted. However, if a planned clinical use requires a sEV preparation enriched for MSC-derived sEVs and at the same time avoiding of process-related contamination by platelet-derived sEVs, our study demonstrates that the use of EV depl. PL might be a feasible, alternative approach.

Expansion systems have been shown to influence sEV potency (Cao et al., 2020; Kim et al., 2020; Yan and Wu, 2020). 3D systems such as hollow fiber bioreactors are favorable since they represent a more native microenvironment of cells (Almeria et al., 2022) and allow the collection of several liters of CM for large-scale manufacturing of sEVs. MSC expansion with hollow fiber bioreactors such as the Quantum system of Terumo BCT has already been implemented for several cell-based applications (Nold et al., 2013; Rojewski et al., 2013; Hanley et al., 2014; Lechanteur et al., 2014; Barckhausen et al., 2016; Haack-Sorensen et al., 2016; Lambrechts et al., 2016; Haack-Sorensen et al., 2018; Mizukami et al., 2018; Frank et al., 2019; Mennan et al., 2019; Lisini et al., 2020; Vymetalova et al., 2020; Cocce et al., 2021; Haberle et al., 2021; Mendt et al., 2021). However, only few studies investigated the use of hollow fiber bioreactor-based systems for the production of MSC-derived sEVs (Mendt et al., 2018; Cao et al., 2020; Yan and Wu, 2020; Gobin et al., 2021; Bellio et al., 2022). In addition, Witwer et al. highlighted the urgent need to investigate the impact of bioreactor-based production processes on sEV characteristics (Witwer et al., 2019).

We showed (to our best knowledge) for the first time a systematic comparison of isolation methods for sEVs generated with the Quantum hollow fiber bioreactor and a CellSTACK-based conventional cell culture system already approved for clinical applications (Gjerde et al., 2018; Gomez-Barrena et al., 2019; Rojewski et al., 2019; Gomez-Barrena et al., 2020; Gomez-Barrena et al., 2021). Surface antigen expression patterns displayed less positive sEVs for known platelet markers such as CD29, CD62P, CD41b, CD42a, and CD31 (Stenberg et al., 1985; Hynes, 1987; Hickey et al., 1989; Newman et al., 1990; Staatz et al., 1991) for the Quantum system, indicating a lower proportion of PL-derived sEVs. This hypothesis is supported by a lower positivity for these markers for MSC-sEVs from αMEM+8% EV depl. PL cultures as compared to PL-sEVs from media. Before media is added to the Quantum system, it has to cross a sterile filter barrier (pore size of 0.2 µm) during filling of media bags. Thus, PL-derived sEVs could have been partially lost in the filter pores, suggesting the Quantum system being helpful in reducing contaminating PL-sEVs and enriching for MSC-derived sEVs. CD49e, also known as integrin α-5, is equally expressed by MSC- and PL-derived sEVs, as shown by similar expression levels for sEVs from media and αMEM+8% EV depl. PL cultures. As part of the fibronectin receptor (Zhang et al., 1993), the differences we observed for Quantum- and CellSTACK-derived sEVs could be the result of divergent adhesion mechanisms for respective MSCs with CP coating of the surface of the Quantum system in contrast to a tissue culture-treated cell culture surface of CellSTACKs.

Although lower protein and particle concentrations were obtained for the isolation of Quantum-derived sEVs by CFF with UC (method IV), as compared to the gold standard method DC with UC (method I), similar expression levels of Flot-1, CD63, and CD81 could provide evidence for equal sEV quantity. This was further reinforced by higher particles to protein ratio indicating higher sEV purity for method IV as proposed by Webber and Clayton (2013). Actual sEV quantity is often overestimated as direct sEV quantification methods are still lacking and quantification by protein and particle concentrations, respectively, is not specific for sEVs (Witwer et al., 2019). Therefore, alternative approaches such as quantification by lipid concentrations (Osteikoetxea et al., 2015; Visnovitz et al., 2019) or fluorescence-based NTA were assumed to be more accurate (Desgeorges et al., 2020).

Other studies described higher sEV quantity for alternative hollow fiber-based expansion systems as compared to 2D culture methods (Cao et al., 2020; Yan and Wu, 2020). Although protein and particle concentrations were comparable to sEVs from CellSTACKs in this study, as shown by similar proteins and particles per cell, respectively, overall sEV yield would be increased for the Quantum system due to higher number of harvested cells. In addition, different parameters could be optimized during collection of sEVs with the Quantum system in order to improve sEV yield. A prolonged sEV collection period could enhance sEV quantity by increasing the amount of CM as a starting material for sEV isolation. Mechanical stimuli such as shear forces have been shown to induce the release of sEVs by leading to elevated intracellular levels of calcium ions important for sEV secretion (Taylor et al., 2020; Kang et al., 2022). Therefore, impact of the media flow rate on sEV yield was investigated by Kang et al. showing best results for a flow rate of 1 mL/min in a flat-plate bioreactor (Kang et al., 2022). In our study, flow rates during sEV collection ranged from 0.4 to 1.6 mL/min demanding further investigation of optimal media flow rates for sEV release. Based on our data, we propose an approach using the Quantum hollow fiber bioreactor as a semi-automated large-scale sEV production system in combination with sEV isolation by CFF with UC for large-scale, GMP-grade sEV generation. Translation of this yet laboratory-scale optimized process toward a GMP-compliant manufacturing for clinical applications could be supported by quality by design (QbD) approaches. Critical process parameters and steps could be identified and different quality controls (e.g., microbial or endotoxin testing) should be included in the whole manufacturing process (Yu et al., 2014; Paolini et al., 2022).

Finally, the impact of isolation methods and culture conditions on sEV potency and functionality remains to be elucidated. However, given the broad field of therapeutic applications with each potentially requiring specific sEV characteristics and the major challenges in establishing reproducible and robust potency assays, this will be subject of future investigations (Witwer et al., 2019; Gimona et al., 2021).

## Data Availability

The raw data supporting the conclusions of this article will be made available by the authors, without undue reservation.
