# Supplementary figures and images for: A novel approach for large-scale manufacturing of small extracellular vesicles from bone marrow-derived mesenchymal stromal cells using a hollow fiber bioreactor
Source: Front Bioeng Biotechnol. 2023 Jan 24;11:1107055. doi: 10.3389/fbioe.2023.1107055 (PMC9904364; doi:10.3389/fbioe.2023.1107055)

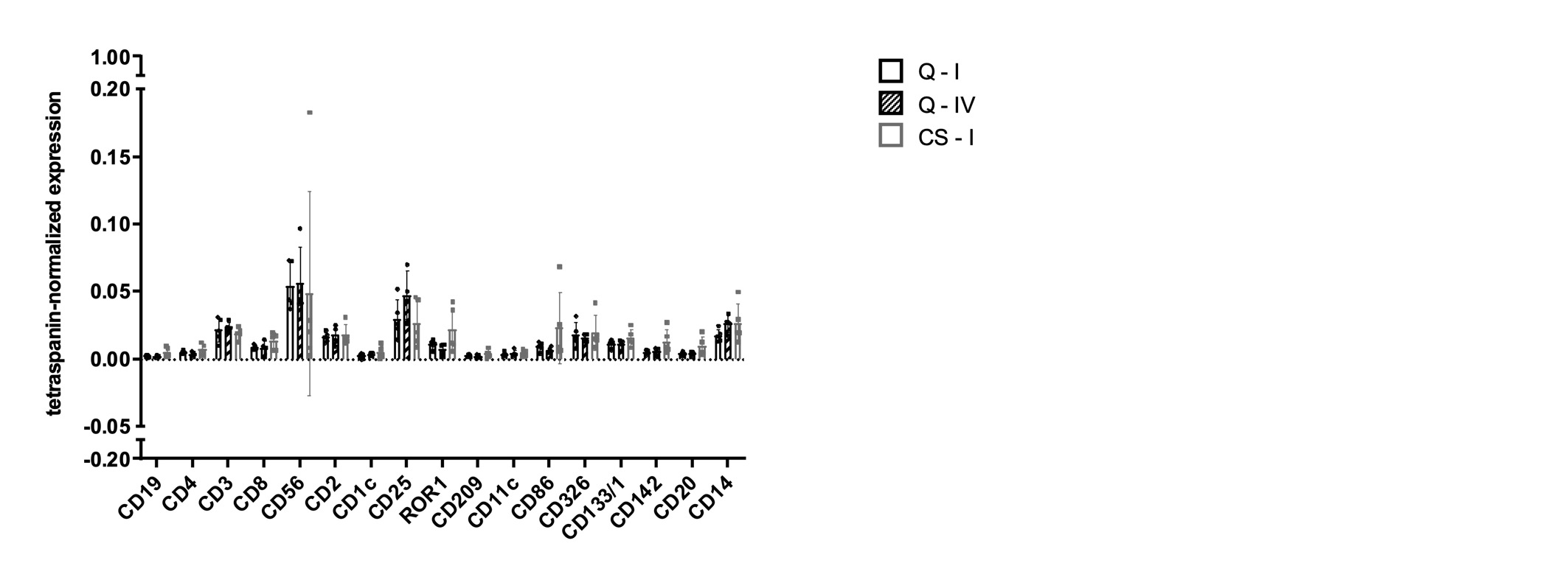

Supplement: Supplementary file 1 [file Image3.JPEG]

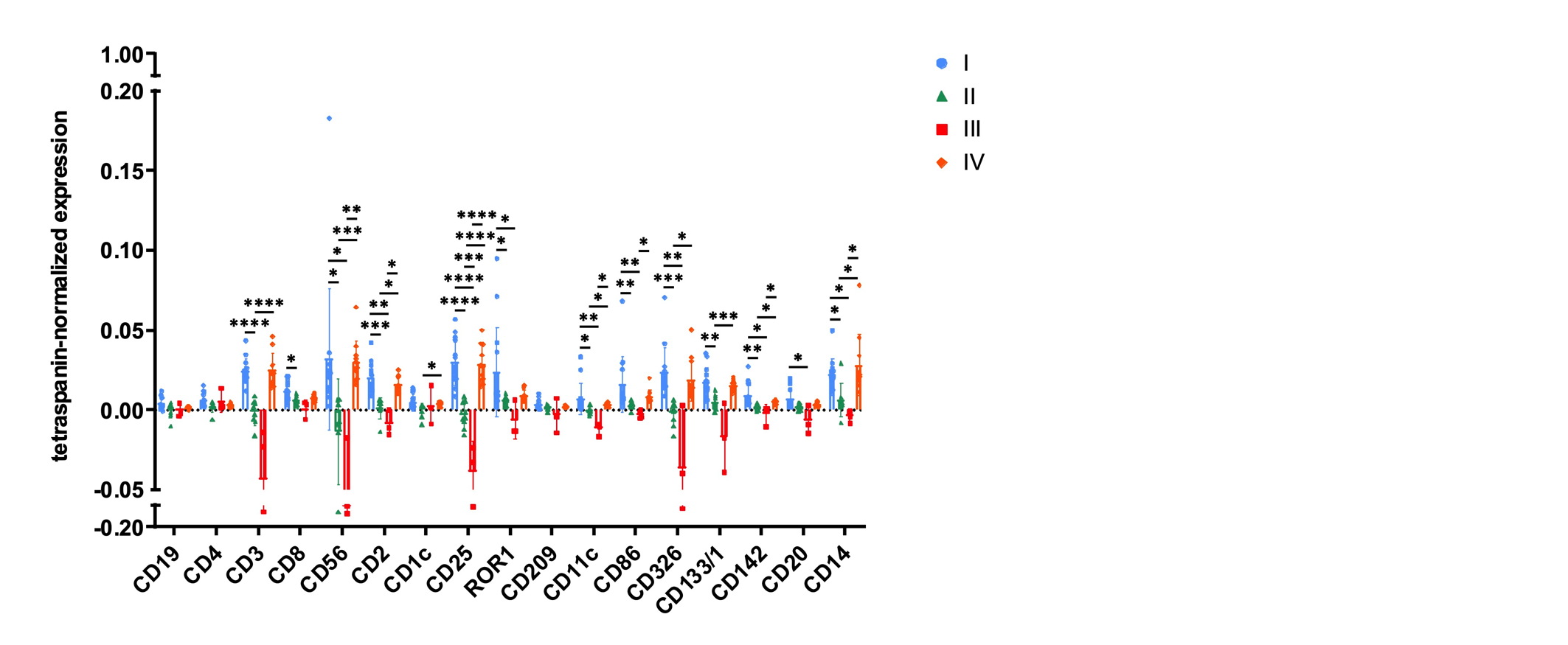

Supplement: Supplementary file 3 [file Image1.JPEG]

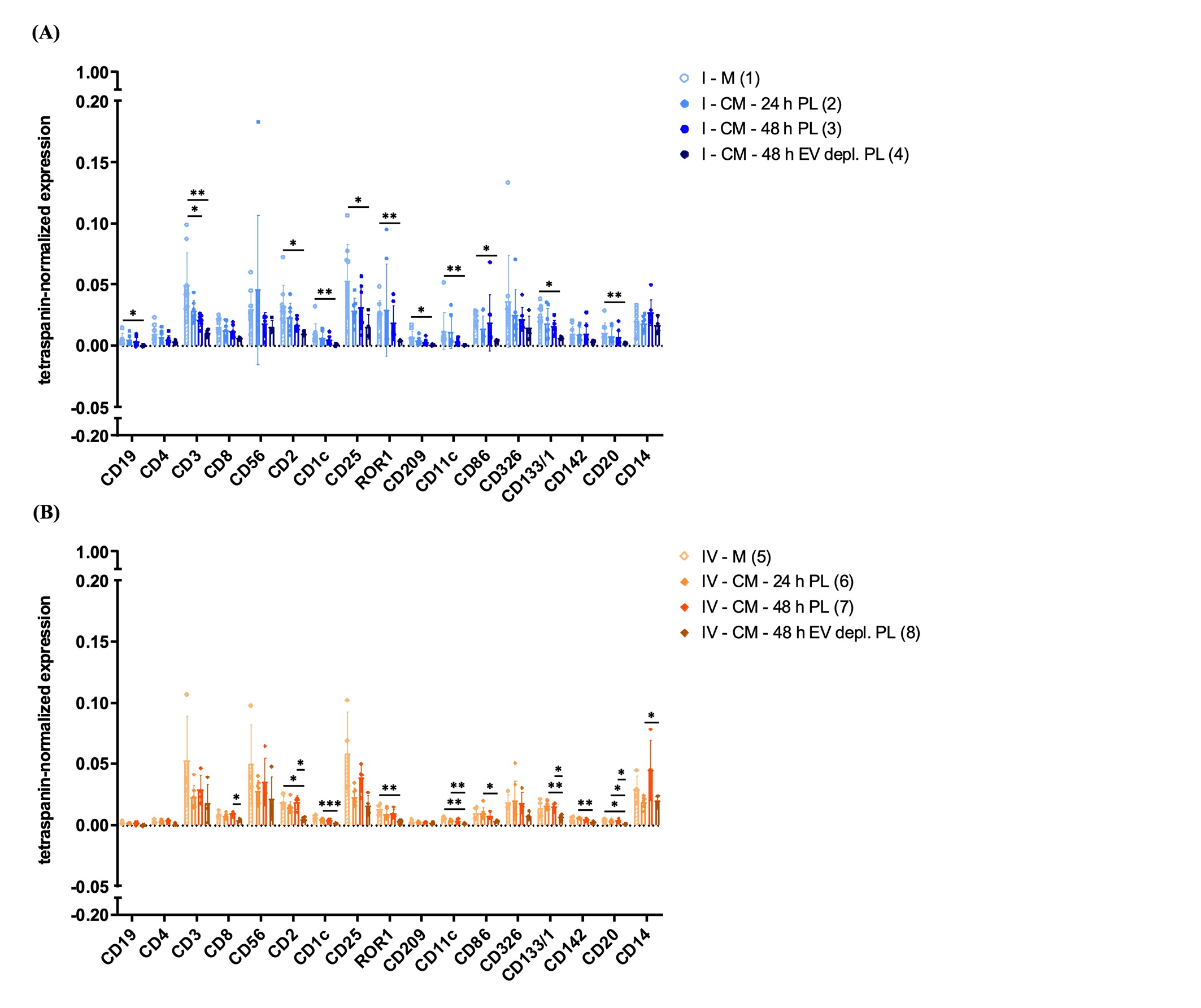

Supplement: Supplementary file 4 [file Image2.JPEG]
