# Supplementary material for: A novel approach for large-scale manufacturing of small extracellular vesicles from bone marrow-derived mesenchymal stromal cells using a hollow fiber bioreactor
Source: Front Bioeng Biotechnol. 2023 Jan 24;11:1107055. doi: 10.3389/fbioe.2023.1107055 (PMC9904364; doi:10.3389/fbioe.2023.1107055)
Supplement: Supplementary file 2 [file Table1.docx]

Supplementary Table 1: Combination of antibodies for flow cytometric characterization of MSCs. MSCs were stained with brilliant violet (BV) 421-, BV786-, fluorescein isothiocyanate (FITC) -, phycoerythrin (PE) -, peridinin chlorophyll (PerCP) –coupled antibodies in six different (1 - 6) staining approaches where number 1 and 4 served as isotype controls (IgG).

| **Number** | **BV421** | **BV786** | **FITC** | **PE** | **PerCP** |
| --- | --- | --- | --- | --- | --- |
| **1** | IgG |  | IgG | IgG | IgG |
| **2** | CD105 |  | HLA DRDPDQ |  |  |
| **3** |  |  | CD90 | CD34 | CD14 |
| **4** |  | IgG |  | IgG |  |
| **5** |  | CD45 |  |  |  |
| **6** |  |  |  | CD73 |  |
